# Effect of paleopolyploidy and allopolyploidy on gene expression in banana

**DOI:** 10.1186/s12864-019-5618-0

**Published:** 2019-03-27

**Authors:** Alberto Cenci, Yann Hueber, Yasmin Zorrilla-Fontanesi, Jelle van Wesemael, Ewaut Kissel, Marie Gislard, Julie Sardos, Rony Swennen, Nicolas Roux, Sebastien Christian Carpentier, Mathieu Rouard

**Affiliations:** 1 Bioversity International, Parc Scientifique Agropolis II, 34397 Montpellier Cedex 05, France; 2 Laboratory of Tropical Crop Improvement, Division of Crop Biotechnics, KU Leuven, B-3001 Leuven, Belgium; 3 MGX-Montpellier GenomiX, Montpellier Genomics and Bioinformatics Facility, F-34396 Montpellier, France; 4 Bioversity International, Willem De Croylaan 42, B-3001 Leuven, Belgium; 5 International Institute of Tropical Agriculture. c/o The Nelson Mandela African Institution for Science and Technology (NM-AIST), P.O. Box 447, Arusha, Tanzania

**Keywords:** Banana, Polyploidy, Paralogs, Genome structure, Transcriptomics

## Abstract

**Background:**

Bananas (*Musa* spp.) are an important crop worldwide. Most modern cultivars resulted from a complex polyploidization history that comprised three whole genome duplications (WGDs) shaping the haploid *Musa* genome, followed by inter- and intra-specific crosses between *Musa acuminata* and *M. balbisiana* (A and B genome, respectively). Unresolved hybridizations finally led to banana diversification into several autotriploid (AAA) and allotriploid cultivars (AAB and ABB). Using transcriptomic data, we investigated the impact of the genome structure on gene expression patterns in roots of 12 different triploid genotypes covering AAA, AAB and ABB subgenome constitutions.

**Results:**

We demonstrate that (i) there are different genome structures, (ii) expression patterns go beyond the predicted genomic groups, and (iii) the proportion of the B genome influences the gene expression.

The presence of the B genome is associated with a higher expression of genes involved in flavonoid biosynthesis, fatty acid metabolism, amino sugar and nucleotide sugar metabolism and oxidative phosphorylation. There are cultivar-specific chromosome regions with biased B:A gene expression ratios that demonstrate homoeologous exchanges (HE) between A and B sub-genomes. In two cultivars, aneuploidy was detected. We identified 3674 genes with a different expression level between allotriploid and autotriploid with ~ 57% having recently duplicated copies (paralogous). We propose a Paralog Inclusive Expression (PIE) analysis that appears to be suitable for genomes still in a downsizing and fractionation process following whole genome duplications. Our approach allows highlighting the genes with a maximum likelihood to affect the plant phenotype.

**Conclusions:**

This study on banana is a good case to investigate the effects of alloploidy in crops. We conclude that allopolyploidy triggered changes in the genome structure of a crop and it clearly influences the gene.

**Electronic supplementary material:**

The online version of this article (10.1186/s12864-019-5618-0) contains supplementary material, which is available to authorized users.

## Background

Bananas (*Musa* spp.), including dessert and cooking types, are monocotyledonous plants of the Zingiberales, one of the major orders of the Commelinidae clade, also comprising Poales (grasses) and Arecales (palms). Bananas are one of the main fruit crops grown worldwide and are critical for food security in many tropical and subtropical countries.

Banana cultivars, which originated from inter- and intra-specific crosses between *Musa acuminata* and *M. balbisiana* (both 2n = 2x = 22), are mainly triploid (2n = 3x = 33), as their genome is composed of three subgenomes, i.e. three sets of 11 chromosomes. Triploidy-induced sterility combined with parthenocarpy resulted in edible fruits without seeds. A first level of cultivar classification is based on the subgenome origin: autotriploid cultivars contain three subgenomes derived from *M. acuminata* (AAA), whereas allotriploid cultivars contain one or two *M. balbisiana*-derived subgenomes (AAB and ABB, respectively). Once triploidy is established in combination with sterility, plant propagation becomes exclusively vegetative. Therefore, differentiation caused by mutation and clonal selection by farmers resulted in new cultivars, which all derived from a common foundation event.

The analysis of the *M. acuminata* reference sequence (A genome) revealed that the ancestral monocot genome underwent three whole genome duplications (WGDs) independent from those that took place in the evolution of other monocot lineages (e.g. Poaceae) [1]. The history of the *Musa* WGDs was reconstructed by collinearity analysis of duplicated genes which were estimated to comprise one-third of the total number of genes [1, 2], while no genome dominance or biased fractionation was detected [2]. Consequently, the banana genome contains a high number of multigene families with abundant recently originated copies (i.e. paralogs) [1, 3], with the post-WGD gene fractionation process still likely ongoing as in other plant species [4]. The analysis of a draft sequence of *M. balbisiana* showed that its genome (B genome) has high degree of homology with a nearly identical number of genes and well-conserved gene-rich regions [5]. However, the two species have a different distribution area and have likely evolved, diverging to better fit their respective environment. For example, differences in codon usage have been detected between the two species [6]. These differences could be useful to understand the genomic constitution of allopolyploids banana cultivars.

So far, expression analyses in allopolyploids have been used for comparisons of gene expression between polyploids and progenitors in coffee and soybean [7, 8], detection of expression bias between paralogs in cotton [9], transcriptomes involved in pathogen defense [10] or in biological processes such as meiosis in *Brassica* [11]. It has also been shown that the coexistence of different genomes in allopolyploid cultivars can induce Homoeologous Exchanges (HE), i.e. recombination between chromosomes contributed by different species taking place when allotriploidy was established. In *Brassica napus*, HEs have been shown to cause changes on expression dosage affecting the phenotype of allotetraploid plants [12]. In banana, it had been suggested that intergenome translocations were common [13, 14] and it has been shown that chromosome pairing between A and B chromosomes at meiosis can occur and translocations between A and B genomes has taken place [15]. However, the relationship between genome structure in allotriploidy and gene expression has not been explored.

In this study, we used data sets obtained from root apexes of 12 banana cultivars covering auto and allotriploids (AAA, AAB and ABB). To investigate the potential impact of the *Musa* B genome on the phenotype, we conducted differential gene expression and performed genome structure analyses taking into account the paleopolyploid nature of the *Musa* genome.

## Results

### Read mapping and single-nucleotide polymorphism (SNP) calling

For this study we first used a published RNAseq dataset [16] on roots of three *Musa* cultivars (two AAA and one ABB (‘Cachaco’)) that we re-mapped on the latest version (v2) of the reference *M. acuminata* sequence (A genome). The number of high-quality reads mapped increased from 81 to 92% by using the new reference, highlighting the improvement on gene annotation in the reference assembly [17]. Subsequently, a second RNAseq dataset was produced with ten cultivars to broaden our observations with a larger representation of allopolyploids including additional five ABB, one AAB and three AAA genomes in addition to *Musa* ABB ‘Cachaco’ (Table 1). In total, we obtained an average of 24 million single-end reads per genotype. Between 13.1 and 52.2 million single-end reads were obtained per biological replicate (23.6 M on average) and a range of 67.1 to 90.1% were uniquely mapped (Additional file 1). The read mapping on the A genome remained quantitatively consistent on the autotriploid and allotriploid cultivars and was not significantly affected by the presence of one or two B genome copies (Additional file 1).

**Table 1:** List of banana cultivars selected for the current transcriptomic study including passport data information

Accession code	Sample name	Group	Subgroup	Geographical origin
ITC0643	Cachaco	ABB	Bluggoe	
ITC0767	Dole	ABB	Bluggoe	
ITC0101	Fougamou1	ABB	Pisang Awak	Gabon
ITC0652	Kluai Tiparot	ABB	Klue Teparod	Thailand
ITC1483	Monthan	ABB	Monthan	India
ITC0123	Simili Radjah	ABB	Peyan	India
ITC1441	Pisang Ceylan	AAB	Mysore	
ITC1122	Gros Michel	AAA	Gros Michel	
ITC1482	Poyo	AAA	Cavendish	
ITC0575	Red Dacca	AAA	Red	
ITC0180	Grande Naine	AAA	Cavendish	
ITC0084	Mbwazirume	AAA	EHAB	Burundi

These mappings were used to identify SNPs in order to assess their allopolyploid chromosome structure.

### Determination of the genomic structure

**Fig. 1:**
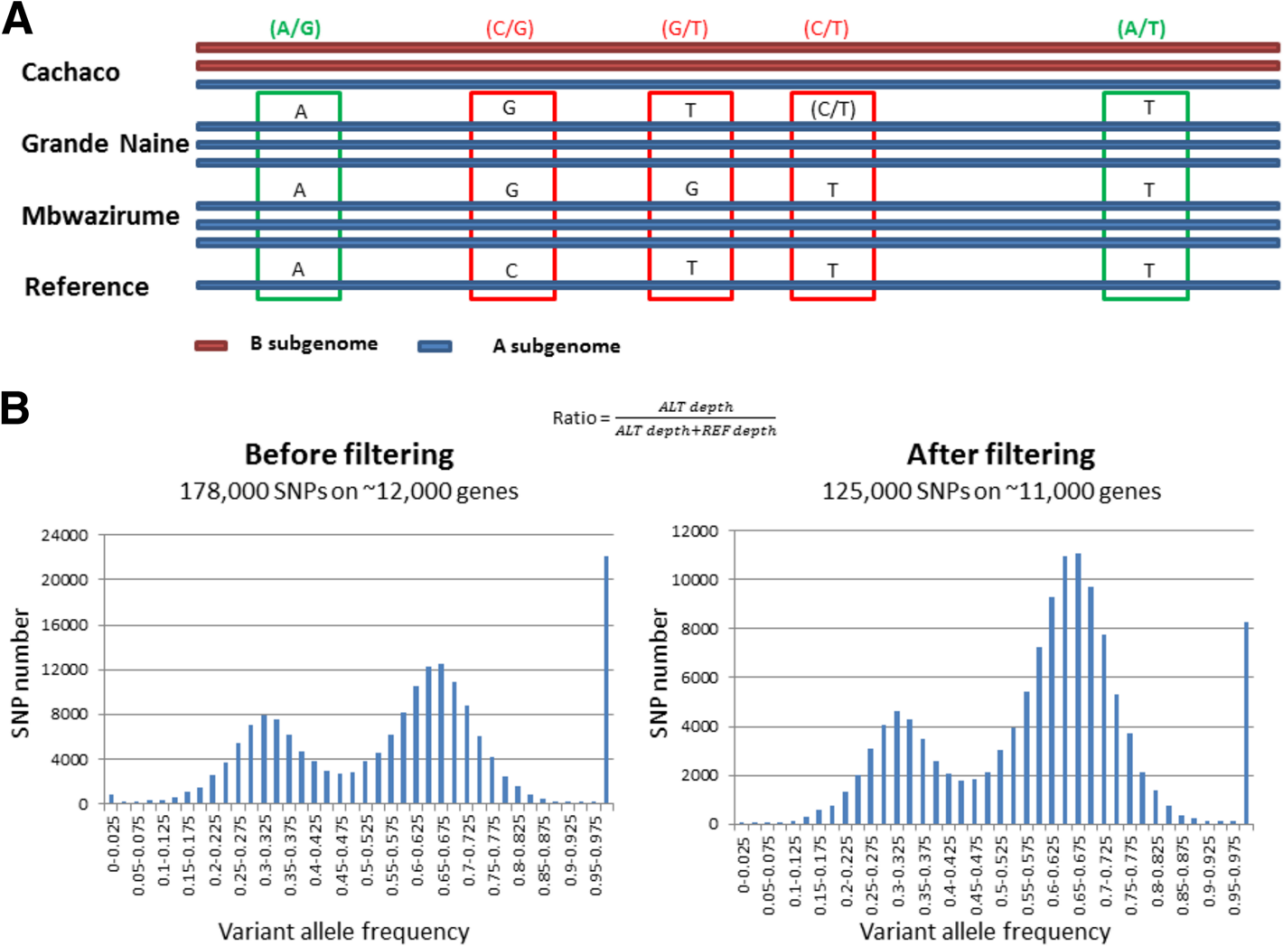
Filtration method to retrieve B specific alleles in ABB cultivars. **a**) Concept of the filtration method to keep only polymorphisms from the B subgenomes. In green the retained SNPs, in red the filtered ones. **b**) Allele frequency distribution of the alternative allele before and after filtration

Around 178,000 ‘Cachaco’ SNPs distributed along the 11 *Musa* chromosomes have been used to estimate the frequency of B genome-specific variants. The SNP positions showing polymorphism within the A reference genome were filtered out by comparing sequence data of ‘Grande Naine’, ‘Mbwazirume’ and the reference sequence of the *Musa* A genome [1] (Fig. 1a). Since the SNP variants specific to the ‘Cachaco’ A subgenome (i.e. not detected in AAA cultivars and A reference genome) are expected to be rare, the remaining 125,000 SNP variants in ‘Cachaco’ were assumed to originate from its two B subgenomes. When the variant SNP allele frequency distributions before and after filtering were compared, both displayed a bimodal distribution (peaks at 1/3 and 2/3), as expected in the presence of three chromosomes, and a histogram bar corresponding to frequency value of 1 (i.e.: 3/3) (Fig. 1b). The reduction in number of SNPs after the filtering varied according to three categories. As expected, the 2/3 peak was less affected by the filtration, supporting the idea that most of these variants are likely B specific (i.e. present in both B subgenomes of ‘Cachaco’). The 1/3 peak lost about half of the SNPs (corresponding to A variants different from the one in the reference A subgenome). The remaining SNPs were, therefore, assumed to be composed of variants specific to one of the two ‘Cachaco’ B subgenomes and of mutations accumulated during the vegetative propagation in one of the three subgenomes. Finally, the 3/3 peak lost about 60% of the SNPs, mostly corresponding to the A reference sequence specific SNPs.

**Fig. 2:**
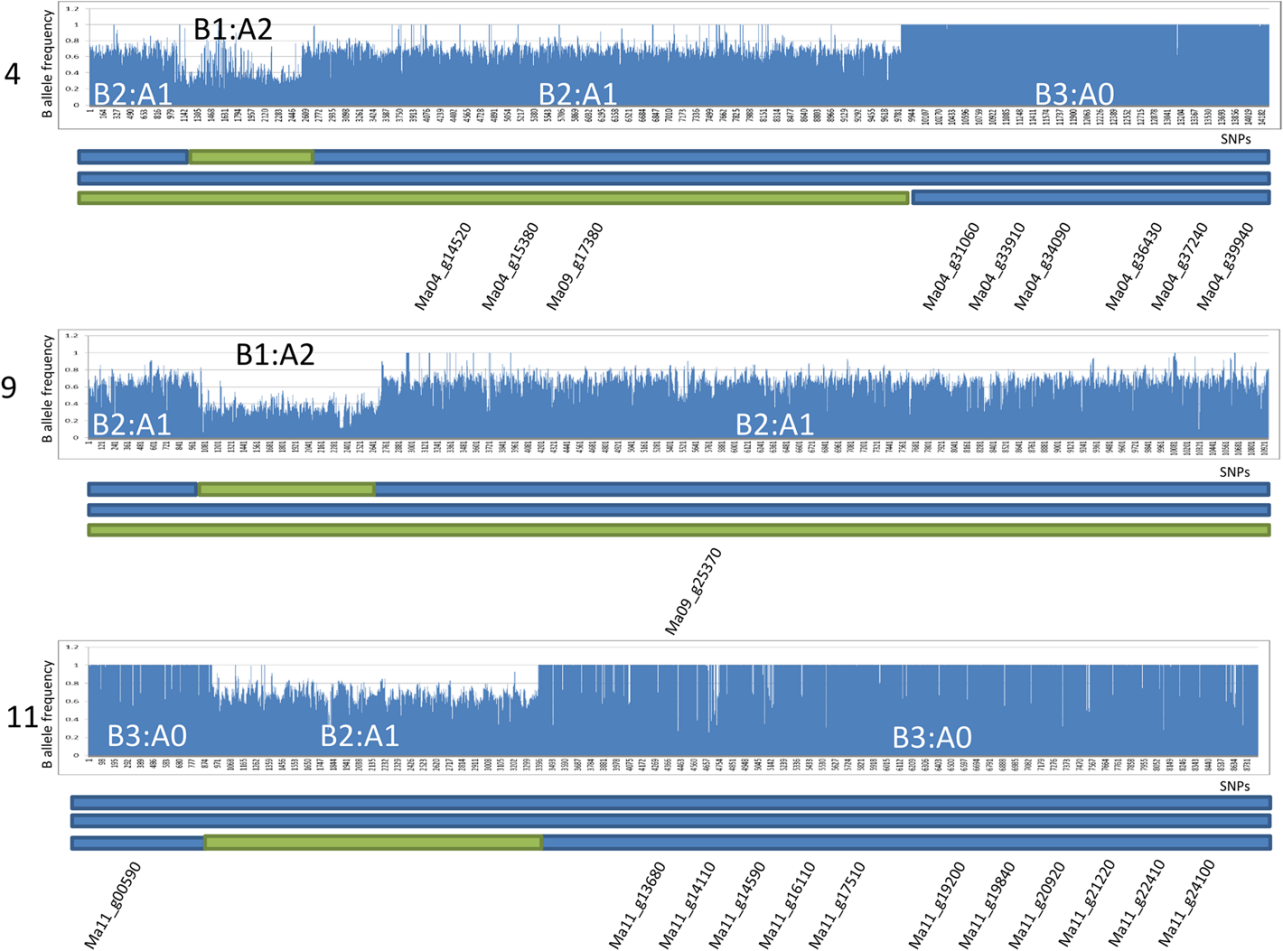
Detected recombination in chromosomes 4, 9 and 11 of Musa ABB Bluggoe ‘Cachaco’. B allele frequency (Y axis) of SNPs ordered according to their position on respective chromosome (X axis) reveals regions deviating from the expected 2/3 (B2:A1) genomic ratio. Recombinant structure inferred (green and blue segment represent A and B subgenomes, respectively) and distribution of DEGs between ‘Cachaco’ and ‘Grande Naine’/‘Mbwazirume’ along the chromosomes

The genome constitution of ‘Cachaco’ being ABB, the B variant frequency distribution along the 11 *Musa* chromosomes was around 67%. However, in two interstitial regions, the frequency of B variants was close to 33% (chromosomes 4 and 9) and in three other large terminal regions, the frequency of B variants was 100% for almost all the SNPs (B3:A0 pattern, in chromosome 4 and at both ends of chromosome 11) (Fig. 2; Additional file 2). In these regions, residual SNPs were observed having a bimodal distribution, with peaks at 33 and 67% (B1:A2 pattern). This indicates the presence of three genomes, excluding the hypothesis of partial A subgenome deletion and, thus, suggesting HE due to recombination. Among the 33,615 genes annotated in the *Musa* chromosomes, 3105 (9.24%) genes of ‘Cachaco’ do not have any A subgenome homeoallele while 861 (2.56%) have a double dose of the A homeoallele.

The same approach was applied to all allotriploid cultivars (Additional file 2). ‘Monthan’ and ‘Dole’ shared all the recombinations detected in ‘Cachaco’; ‘Simili Radjah’ shared some recombination with the ‘Cachaco’, ‘Dole’ and ‘Monthan’ recombination pattern (Additional file 2) and showed an additional interstitial region on chromosome 5, where the genome ratio is B1:A2. ‘Fougamou1’ showed several recombination events, but all were different from those of ‘Cachaco’, ‘Dole’ and ‘Monthan’. ‘Kluai Tiparot’ also had several recombinations different from the ones detected in ‘Cachaco’, ‘Dole’ and ‘Monthan’ and ‘Fougamou1’. However, in ‘Kluai Tiparot’, all recombinations were characterized by a B3:A0 ratio. Finally, in the single AAB cultivar ‘Pisang Ceylan’, some recombinations with both B0:A3 and B2:A1 ratios were detected (Additional file 2).

In two cultivars, aneuploidy was also detected: the whole chromosome 8 is missing in ‘Dole’ and the long arm of ‘Simili Radjah’ chromosome 5 has only two of the three expected allelic doses.

### B genome impact on the transcriptomes of allotriploid cultivars

**Fig. 3:**
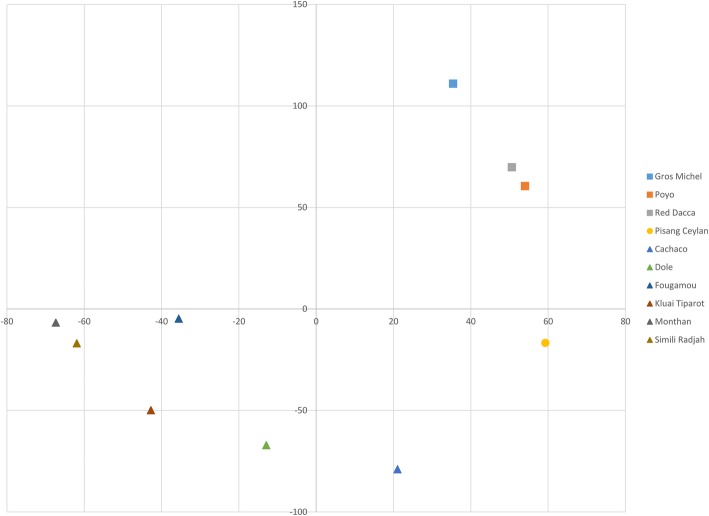
Partial Least Squares (PLS) analysis on 10 cultivars having auto or allopolyploid genome constitution. Squares, circles and triangles indicate genome constitution of the analysed cultivars (AAA, AAB and ABB, respectively). X and Y axes represent PC1 and PC2 variables respectively

A Partial Least Square (PLS) analysis clearly distinguished ABB from AAA cultivars, whereas the unique AAB cultivar ‘Pisang Ceylan’ had an intermediate position (Fig. 3).

**Fig. 4:**
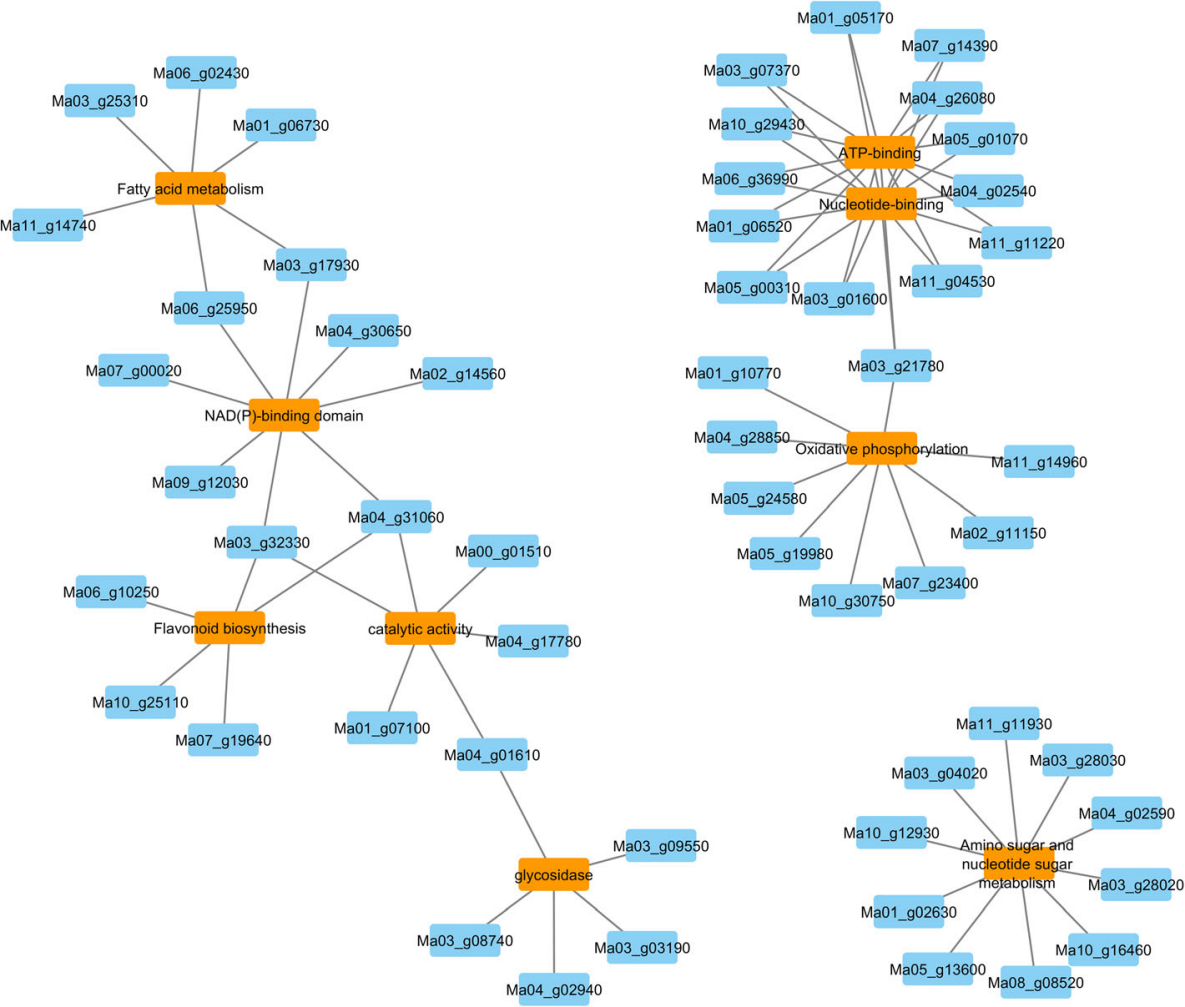
Pathways identified by enrichment analysis on gene set. Pathways are indicated in orange squares and gene id are represented by the blue squares

Genes with a higher expression in the cultivars containing the B genome were enriched in the following pathways: flavonoid biosynthesis, fatty acid metabolism, amino sugar and nucleotide sugar metabolisms and oxidative phosphorylation (Fig. 4).

### Expression analysis in a paleopolyploid genome

A set of 3674 genes was found significantly differentially expressed between ‘Cachaco’ and both AAA cultivars, ‘Grande Naine’ and ‘Mbwazirume’ (Additional file 3). About 57% of the differentially expressed genes (DEGs) were identified as part of multigene families as unique or multiple representatives (Additional file 4). Among these genes, 541 (14.73%) were located in the B3:A0 regions, i.e. they have only the B genome homeoallele, whereas 33 (0.90%) were in the region with double dose of A homeoalleles.

**Fig. 5:**
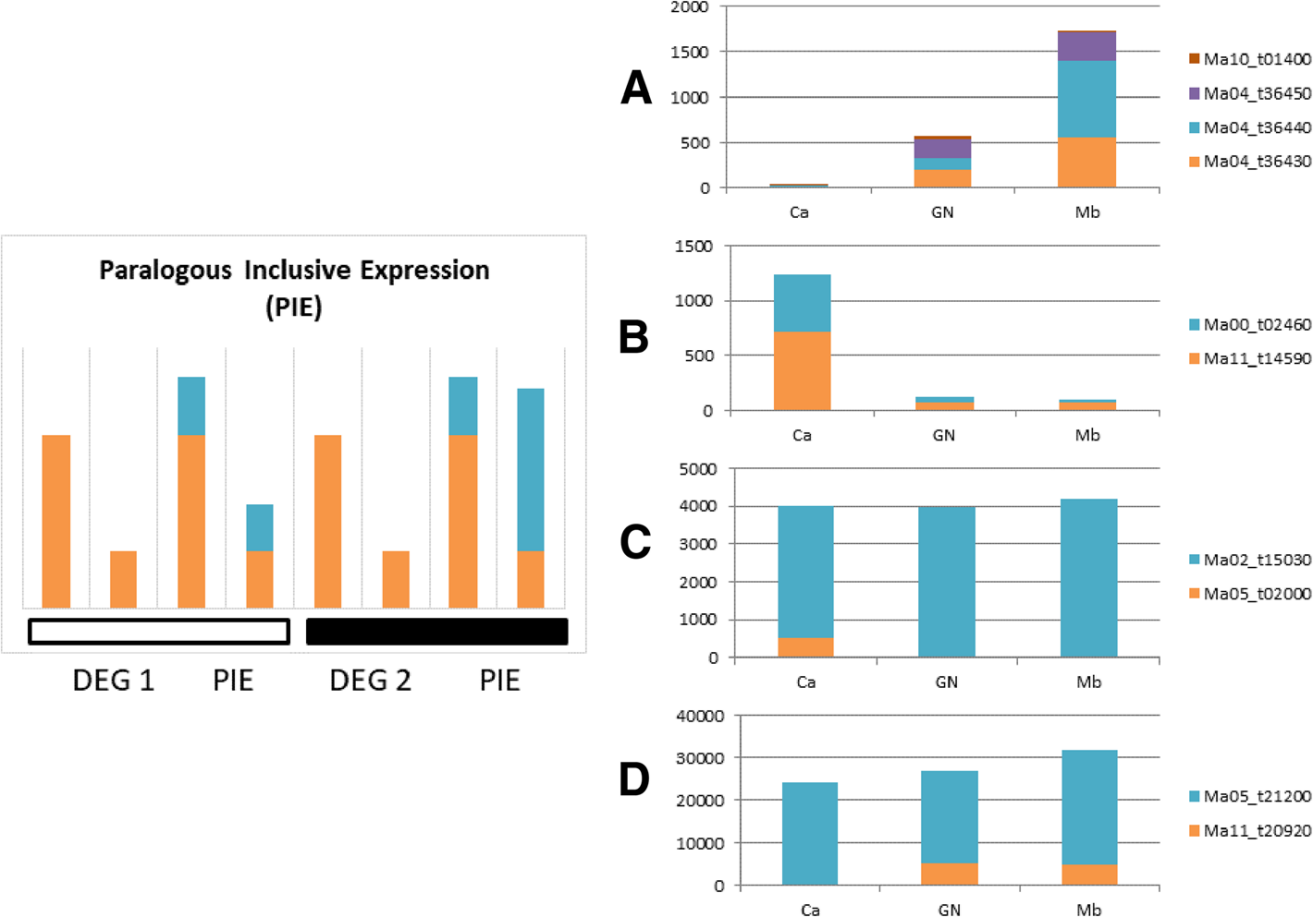
Principle of the Paralogous Inclusive Expression (PIE) and contrasted paralog expression for DEGs in ‘Cachaco’ (Ca) compared to ‘Grande Naine’ (GN) or ‘Mbwazirume’ (Mb). On the left, comparison between DEGs and global expression including paralog expression (PIE). The white bar illustrates an example where DEGs is considered as promising because the global differential expression remains significant. The example with the black bar is discarded as the global expression is comparable between the two compared conditions. On the right, in orange the expression of the genes differentially expressed (normalized read count, Y axis), in other color(s) the expression of respective paralog(s). **a** and **b** are examples for genes whose paralog expression does not invalidate the differential expression of DEG; **c** and **d** are examples of genes whose paralog expression overwhelmed the DEG expression

**Fig. 6:**
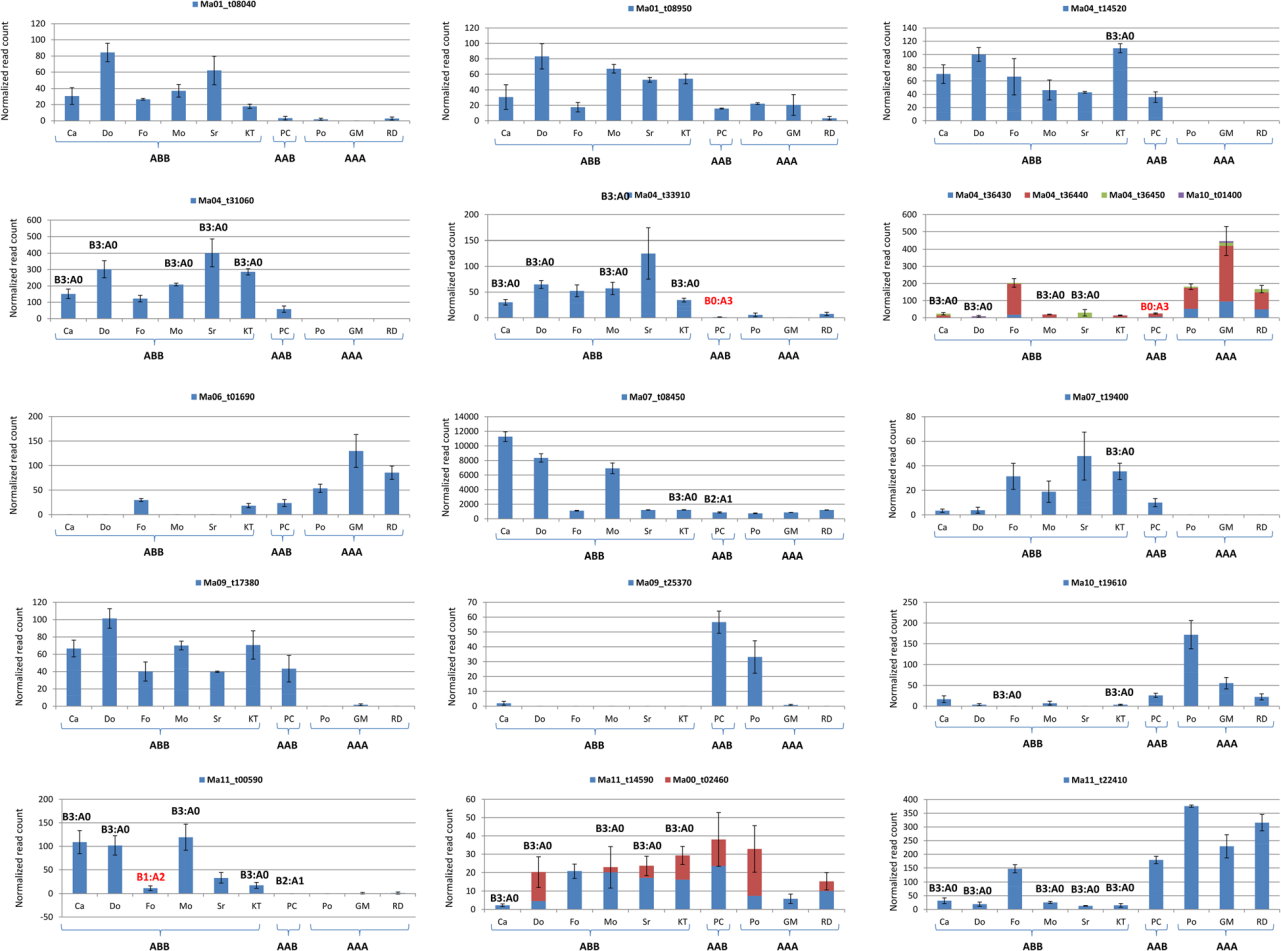
Histograms representing the paralog included expression (normalized read count, Y axis) of 15 differentially expressed genes in cultivar having different genomic constitution: **ABB** (‘Cachaco’ (Ca), ‘Dole’ (Do), ‘Fougamou’ (Fo), ‘Monthan’ (Mo), ‘Simili Radjah’ (SR), and ‘Kluai Tiparot’ (KT)), **AAB** (‘Pisang Ceylan’ (PC)) and **AAA** (‘Poyo’ (Po), ‘Gros Michel’ (GM) and ‘Red Dacca’ (RD)). Blue color represents expression level of DEG, other colors expression level of respective paralogs. The actual homoeologous ratio is indicated for the genes lying in deviating regions in respective cultivars: the increase of B or A homeoalleles is indicated in black or red, respectively

Due to the high proportion of DEGs belonging to multicopy families, a subset of 54 DEGs was selected for manual curation to gain insight into their potential impact on the phenotype (Additional file 5). One-third of those genes (18) were located in the B3:A0 regions, whereas none were located in the B1:A2 regions. Based on paralogous identification, eight belonged to multicopy (> 10) tandem repeated clusters and were no longer analyzed. Among the remaining 46 DEGs, 13 did not have any paralog in the *Musa* genome and 1–9 paralogs were found for the other 34 (Additional file 5). On those 34, a differential expression analysis called Paralog Inclusive Expression (PIE) analysis was performed to take into account the relative expression of the DEG and its lineage specific paralogs. Among them, the expression of only two genes is not overruled by paralogs: 1) Ma04_g36430, whose two of the three found paralogs (being the tandemly duplicated copies Ma04_g36440 and Ma04_g36450) have a consistent expression pattern with the sampled gene (i.e. no expression in ‘Cachaco’ but expression in both AAA cultivars), whereas no or very low expression was detected for the third paralog (Ma10_g01400) (Fig. 5a); 2) Ma11_g14590 whose unique paralog (Ma00_t02460) has consistent expression with Ma11_g14590 (Fig. 5b). For the remaining 32 DEGs, the paralog expression was globally higher than the sampled gene, overruling or reducing the impact of the expression difference between ‘Cachaco’ and AAA cultivars (Ma05_g02000 and Ma11_g20920 as example in Fig. 5c and d; Additional file 6). In the larger dataset including six ABB and three AAA cultivars, differential expression of almost all the 15 validated DEGs, over- or under-expressed in ‘Cachaco’ vs AAA cultivars, was confirmed to be associated with the presence of the B genome (Fig. 6).

In the second dataset, DEGs were identified between each allopolyploid and each of the three AAA genotypes. For each allopolyploid, DEGs detected in all the three comparisons were selected (Table 2). In ‘Pisang Ceylan’ (AAB) a lower number of DEGs was detected (131), whereas among the ABB cultivars the detected DEGs spanned between 547 and 1661 (Table 2). Moreover, the DEGs in chromosome regions showing a deviating genome contribution ratio were counted (Table 2) and similarly to ‘Cachaco’ all the regions with a B3:A0 structure in ABB genotypes (and with B2:A1 in AAB genotype) showed a significant excess of DEGs compared to a random distribution. In the regions where the B contribution was lower than expected (i.e. B1:A2 and B0:A3 in ABB and AAB, respectively) the number of DEGs was globally under-represented (Table 2).

**Table 2:** Over-representation of DEGs (allo- vs autotriploids) in recombined regions of allotriploids

	Genome	B3:A0 regions			B1:A2 regions		
Genotype	DEGs	Genes	DEGs	*P* value	Genes	DEGs	*P* value
Cachaco	987 (0.03)	3105 (0.09)	168 (0.17)	3.0^e-17^	861 (0.03)	12 (0.01)	< 0.01
Dole*	577 (0.02)	3105 (0.10)	111 (0.14)	< 0.01	861 (0.03)	8 (0.01)	< 0.01
Fougamou	823 (0.02)	3483 (0.10)	188 (0.23)	7.1^e-32^	1226 (0.04)	11 (0.01)	< 0.01
Kluai Tiparot	1661 (0.05)	22,105 (0.66)	1249 (0.75)	5.29^e-16^	N/A	N/A	N/A
Monthan	558 (0.02)	3105 (0.09)	102 (0.17)	1.11^e-11^	861 (0.03)	4 (0.01)	< 0.01
Simili Radjah^a^	547 (0.02)	1847 (0.06)	97 (0.16)	1.05^e-25^	393 (0.01)	1 (0.00)	< 0.05
	Genome	B2:A1 regions			B0:A3 regions		
Genotype	DEG	Genes	DEGs	*P* value	Genes	DEGs	*P* value
Pisang Ceylan	131 (0.004)	3116 (0.09)	28 (0.21)	2.8^e-06^	803 (0.02)	2 (0.002)	Not significant

^a^Chromosome 8 and 5 data were excluded from Dole and Simili Radjah, respectively

Gene and DEG numbers (between brackets their ratio with the 35,276 genes annotated in the V2 Musa sequence) were compared in regions with deviating subgenome ratios

## Discussion

### Homoeologous exchanges occurred between a and B genomes

Polyploidy is known to induce changes in genome structure and gene expression [12] and banana is no exception [18]. The structure of the allotriploid genomes studied here is not the mere sum of 11 A chromosomes from *M. acuminata* and 22 B chromosomes from *M. balbisiana*. The observation of chromosome regions showing deviation from the expected 2B:1A ratio (3B:A0 and 1B:2A, according to the region), or, in the case of ‘Pisang Ceylan’ (AAB) from the expected 1B:2A ratio (2B:1A or 0B:3A) implies that recombinant events left their tracks in the allotriploid genomes. It was already hypothesized that most, if not all, banana cultivars have genomes consisting of different proportions of A- and B-genome chromosomes and/or recombinant chromosomes based on chromosomal, nuclear and cytoplasmic DNA and protein data [13]; this was also recently reported for a few cultivars [19].

Since the triploid cultivars are sterile and vegetatively-propagated, it is likely that the detected recombinations originated during the formation of the allotriploid genotypes and were fixed by vegetative propagation. Due to their obliged clonal nature and from an evolutionary point of view, the triploid bananas should be considered as hybrid lineages, even if allotriploid bananas share some features with allopolyploid species (e.g. fixed heterozygosity or inter-genomic interactions). The presence of regions with deviating homeoallele contributions could impact the phenotypes, in particular for the regions 3B:0A (or 0B:3A), where the missing A (or B) genome regions induce a localized lack of inter-genomic heterozygosity and highlights the possible expression differences between the A and B subgenomes. For instance, around 9% of the *Musa* annotated genes lie in genomic regions where the ‘Cachaco’ genome ratio is 3B:A0, but, when only the DEGs between ‘Cachaco’ and both AAA cultivars were considered (3674 genes), the 3B:A0 gene fraction is significantly higher (14.7%, *p* < 2.1^2e-36^ in first dataset and results reported in Table 2 for the second one). We can conclude that the substitution of A homeoalleles increases the probability of significant changes in gene expression and, consequently, in phenotype. Similar dosage-dependent effects on expression of genes in genomic regions that underwent homoeologous exchanges were already observed in *Brassica napus* [12].

### Expression differences between Allo- and autotriploid cultivars are mainly influenced by the presence/absence of the B genome

We aimed to understand the impact of the B subgenome presence on the total gene expression in allopolyploids. The general expression level is altered between the 3 genomic groups (Fig. 3). This confirms the impact of the B genome presence on the gene expression. The presence of the B genome in the roots leads to a higher expression of genes involved in pathways like flavonoid biosynthesis, fatty acid metabolism, amino sugar and nucleotide sugar metabolism and oxidative phosphorylation. The biological functions of flavonoids are linked to their potential cytotoxicity and their capacity to interact with enzymes through protein complexation. Some flavonoids provide stress protection, for example, acting as scavengers of free radicals such as reactive oxygen species (ROS), as well as chelating metals that generate ROS via the Fenton reaction [20]. Roots are for their energy completely dependent on the carbohydrates they receive from the source organs. The preferred way to generate the energy is via metabolizing carbohydrates and fatty acids via oxidative phosphorylation. Fatty acid metabolism, ATP binding, catalytic properties and amino and nucleotide sugar metabolism are general important pathways for root growth & development.

### See the wood for the trees: the role of paralogous genes

Banana has a complex paleopolyploid genome with multiple paralogs. Three WGDs were inferred in the evolutionary history of the banana (haploid) genome. The two more recent and “almost simultaneous” WGDs (α and β) have left more than one-third of genes in multiple copies [1, 2]. WGDs are common and recurrent evolutionary phenomena in plants [21, 22] and evolution by gene duplication is understood to be an important source of phenotypic novelties [23]. For instance, neo-functionalization (i.e. functional divergence of gene copies) or sub-functionalization (space-temporal repartition of the gene function) could give evolutionary advantages. However, at genomic scale, this duplicated status is unstable and tends to evolve towards a diploid status by structural recombinations and by a genome downsizing or fractionation (i.e. loss of duplicated genes that follows the WGDs) [4, 24]. This fragmentation can be a long process that involves the removal of duplicated and functionally redundant genes by accumulation of mutations in the additional copies. In most cases, those mutations (including partial or total deletion) compromise the function of the coded protein. The high number of duplicated genes in the *Musa* genome [1], the higher number of *Musa* gene family members in well-studied gene families when compared to other species [3, 25] and the comparative genome wide analysis of duplicated genes in a large sample of angiosperms [26] (Fig. 4), suggests that the fragmentation process in the *Musa* genome is still ongoing which makes the proportion of paralogs with redundant function likely high.

In our analyses, we found paralogs for the large majority of DEGs selected in ABB/AAA comparisons. The presence of paralogous gene copies (potentially being functionally redundant), makes it trickier to interpret differential gene expression results. Considering an enzymatic function ensured by more than one gene, the impact of regulation changes needs to be considered for all the gene copies (Fig. 5).

A panel of genes showing highly significant different expression between ‘Cachaco’ (ABB) and AAA cultivars was analyzed to identify paralogs and to determine the expression changes and the possible impact on the phenotype by a Paralog Inclusive Expression (PIE) analysis (Additional file 5). In a subset of 46 DEGs between ‘Cachaco’ and two AAA cultivars, 32 DEGs have paralogs with expression that overwhelms the differences observed in the considered genes, with a likely negligible impact on the phenotype. Fourteen genes could potentially have an influence on the phenotype as 13 of them have no paralogs (Additional file 5) and the other two have paralogs whose expression level is very reduced or consistent with the analyzed DEGs. Interestingly, these genes were selected among the most significant DEGs and were detected in almost all ABB genotypes.

For some genes, these copies may be still diverging in their expression after the *M. acuminata* and *M. balbisiana* lineage separation. Indeed, in a given genome a gene can have recently lost (or strongly limited) its expression without consequences for the phenotype due to the presence of redundant paralogs, and be maintained in the other genome, inducing a significant different expression between A and B homeoalleles but with no obvious effect on the phenotype. However, the impact of several enriched genes in the same pathway or function gives confidence of their potential effect on the phenotype.

## Conclusions

We observed marked differences between auto and allotriploid transcriptomes, the most significant DEGs often being correlated with the presence or absence of the B subgenome. There is a large variability among the ABB cultivars due to the different genomic structures detected in the sampled cultivars (homoeologous exchanges), which testify independent foundation events for the ABB subgroup and, likely, with different A and B genome contributions.

Since the occurrence of WGDs is one of most frequent evolutionary events in plants, the occurrence of paralogs could be generalized for all plant species. However, the impact of duplicated and functionally redundant genes is inversely correlated with the time elapsed from the most recent WGD and with the specific evolution rate. In fact, the progress of the fractionation and the neo- and sub-functionalization of duplicated genes reduce the occurrence of functionally redundant genes and, consequently, increase the probability that an observed expression difference impacts the phenotype.

The presence of multi-copy genes adds to the background noise in whole genome expression analysis, thus increasing the need for expert analyses to draw correct conclusions. For species having genomes with a high number of paralogs, an automatic pipeline for paralogous detection combined with the evaluation of regulation changes in gene multi-copy context would be relevant.

## Methods

### Plant material and growth conditions

Twelve varieties belonging to AAA, AAB and ABB subgroups were supplied by the Bioversity International *Musa* Germplasm Transit Centre (ITC), hosted at KU Leuven, Belgium (Table 1). Plants were grown as described by Zorrilla-Fontanesi et al. (2016), using a Bronson incubator (Bronson Incubator Services B.V., Nieuwkuijk, Netherlands). In vitro plants were grown for 21 days, after which root tips (4–5 cm long) were collected and snap frozen separately in liquid nitrogen and stored at − 80 °C.

### RNA extraction and cDNA library sequencing

Total RNA was extracted as described in [27]. For cDNA library construction, RNA integrity was checked by the ExperionTM (BIO-RAD Laboratories, Inc. USA; RQI > 9.4) and BioAnalyzer (Agilent; RIN > 7.8). In total, 9 and 30 RNA samples (for the first and second experiment, respectively) were isolated from 3 biological replicates per genotype. Multiplex sequencing on an Illumina HiSeq2000 was performed as 100 bp, single reads at the Montpellier GenomiX facility as described in Zorrilla-Fontanesi et al. (2016).

### RNA-Seq filtering, mapping and SNP calling

RNA-Seq reads were quality-filtered using the Illumina purity filter. Quality of reads was checked using FastQC v0.11.2 [28]. Reads were cleaned to remove adapter sequences and low-quality ends (phred score > 20) with Cutadapt v2.7.9 (Martin, 2011). After trimming, reads inferior to 30 bp were discarded. Reads were then aligned against the reference *M. acuminata* genome (DH Pahang) v2 [17, 29] using the splice junction mapper for RNA-Seq STAR v2.5.0 [30] in a 2-pass mode with default parameters. Read groups were added for each alignment file (SAM). Reads were then split using SplitNCigarReads and locally realigned with IndelRealigner from GATK (Genome Analysis ToolKit) v3.4 [31]. SNPs were called on uniquely mapped reads with UnifiedGenotyper from GATK (ploidy parameter set to 3). The variant annotation and effect prediction was performed with SnpEff v4.1 [32]. An overview of the bioinformatics pipeline is shown in Additional file 7.

### Allotriploid genomic structure

Our dataset of genotypes ‘Mbwazirume’, ‘Cachaco’ and ‘Grande Naine’ was used as a starting point for further analysis. All the reads were realigned to the newest version of the *Musa* genome (derived from the genomic sequence of *M. acuminata* DH Pahang v2 (Martin et al., 2016)).

The allotriploid genomic structure was verified by checking the 2/3 expected contribution of B homeoallele variants (1/3 for ‘Pisang Ceylan’, AAB) of SNPs in the expressed genes along the 11 chromosomes. In order to reduce the bias introduced by SNPs due to the A sequence polymorphism, only SNP positions monomorphic in available A genomes (DH Pahang and RNA-Seq data of AAA cultivars ‘Grande Naine’ and ‘Mbwazirume’) were taken into account. At each retained SNP position, the alternative nucleotide to the reference variant was considered the B genome variant (Fig. 1a).

### Multivariate analysis

To find genotype specific gene expression patterns, a Partial Least Squared analysis was performed with the NIPALS algorithm on the normalized read counts (Statistica 13, Non Sigma) of the larger sample dataset. 35,307 annotated genes were considered as X variables and the number of B chromosomes as Y variables (0, 1, 2). Normalized reads were subjected to ANOVA analysis and Benjamini correction (33,667 variables).

### Gene enrichment

To analyse possible gene enrichment, the Benjamini corrected variables were selected according to their ABB or AAA profile and their accession numbers were converted to entrez gene IDs using the conversion tool (http://banana-genome-hub.southgreen.fr/convert). Entrez gene IDs were submitted to DAVID [33]. Significant enrichments (EASE threshold 0.1) were exported and visualized using Cytoscape [34].

### Differential gene expression analyses

The number of reads in genes were counted with HTSeq-count [35] using the corresponding gene annotation file and the “union” mode. Differential gene expression was evaluated using edgeR v3.12.0 [36] in the R statistical environment (R Core Team, 2013). The *p*-value was adjusted for multiple testing by controlling the false discovery rate (FDR) at ≤5%. Data were normalized using RLE [37].

A subset of DEGs was created for manual curation processes. We first sorted all DEGs based on their respective p-value for ‘Cachaco’ vs ‘Mbwazirume’ and ‘Cachaco’ vs ‘Grande Naine’ (*n* = 3) (Additional file 3). Then, we considered arbitrarily the DEGs in the first 150 positions of both rankings to select the most highly differently expressed genes common to both.

### Paralog detection and paralog inclusive expression (PIE) analysis

Identification of *Musa* specific paralogs on the whole set of differentially expressed genes (DEGs) was performed using Orthofinder v2.2.1 with protein sequences from *Musa acuminata* v2, *Oryza sativa v7*, *Arabidopsis thaliana v10* and from *Vitis vinifera v1*. The number of paralogs was defined by counting the number of gene occurrence of *Musa* within each orthogroup.

The presence of lineage specific paralogs (in-paralogs) in the *Musa* genome for a limited number of selected DEGs was verified by BLASTp analysis on Non-redundant protein database at NCBI [38]. *Musa* genes, having higher scores than genes from any other species, were considered in-paralogs. Even if orthology/paralogy is defined based on phylogenetic analysis, we considered our simplified approach based on sequence similarity appropriate to find paralogs and the possible bias due to lack of identification of paralogs with high sequence divergence as negligible. The same approach was used in [39]). When one or more paralogs were found, normalized counting of each paralog was added to the one of the respective DEG and paralog inclusive expression was compared among samples.

## Additional files

Additional file 1: Sequencing and genome mapping statistics for the 12 genotypes considered in the study. Since ‘Cachaco’ was present in both the experiments, it was represented twice. (PNG 1026 kb)

Additional file 2: Chromosome intervals (defined on genes coordinates) showing B:A ratio deviating from the expected genomic constitution. CDM indicates the three cultivars sharing the same pattern of deviating regions (‘Cachaco’, ‘Dole’ and ‘Monthan’). (DOCX 16 kb)

Additional file 3: List of 3674 genes showing different expression levels between ‘Cachaco’ (ABB) and both AAA cultivars (‘Grande Naine’ and ‘Mbwazirume’). LogFC, logCPM, *p*-value and FDR are reported for both comparisons. (XLSX 513 kb)

Additional file 4: Distribution of the 3674 DEGs by number of paralogs. (PNG 128 kb)

Additional file 5: Sample of genes differentially expressed in ‘Cachaco’ (ABB) compared to AAA cultivars (‘Mbwazirume’ and ‘Grande Naine’). (DOCX 18 kb)

Additional file 6: Histograms representing the paralog included expression (normalized read count, Y axis) for 58 genes having significant higher expression in ‘Cachaco’ (Ca) than in ‘Grande Naine’ (GN) and ‘Mbwazirume’ (Mb). Blue color represents expression level of DEG, other colors expression level of respective paralogs. (PPTX 146 kb)

Additional file 7: Schematic view of the bioinformatics workflow for differential gene expression and genome structure identification. (PNG 310 kb)

## Abbreviations

DEG: Differentially expressed gene
HE: Homoeologous exchange
PIE: Paralog Inclusive Expression
PLS: Partial Least Square
ROS: Reactive oxygen species
SNP: Single-nucleotide polymorphism
WGD: Whole genome duplication
