# Supplementary figures and images for: Effect of paleopolyploidy and allopolyploidy on gene expression in banana
Source: BMC Genomics. 2019 Mar 27;20:244. doi: 10.1186/s12864-019-5618-0 (PMC6438041; doi:10.1186/s12864-019-5618-0)

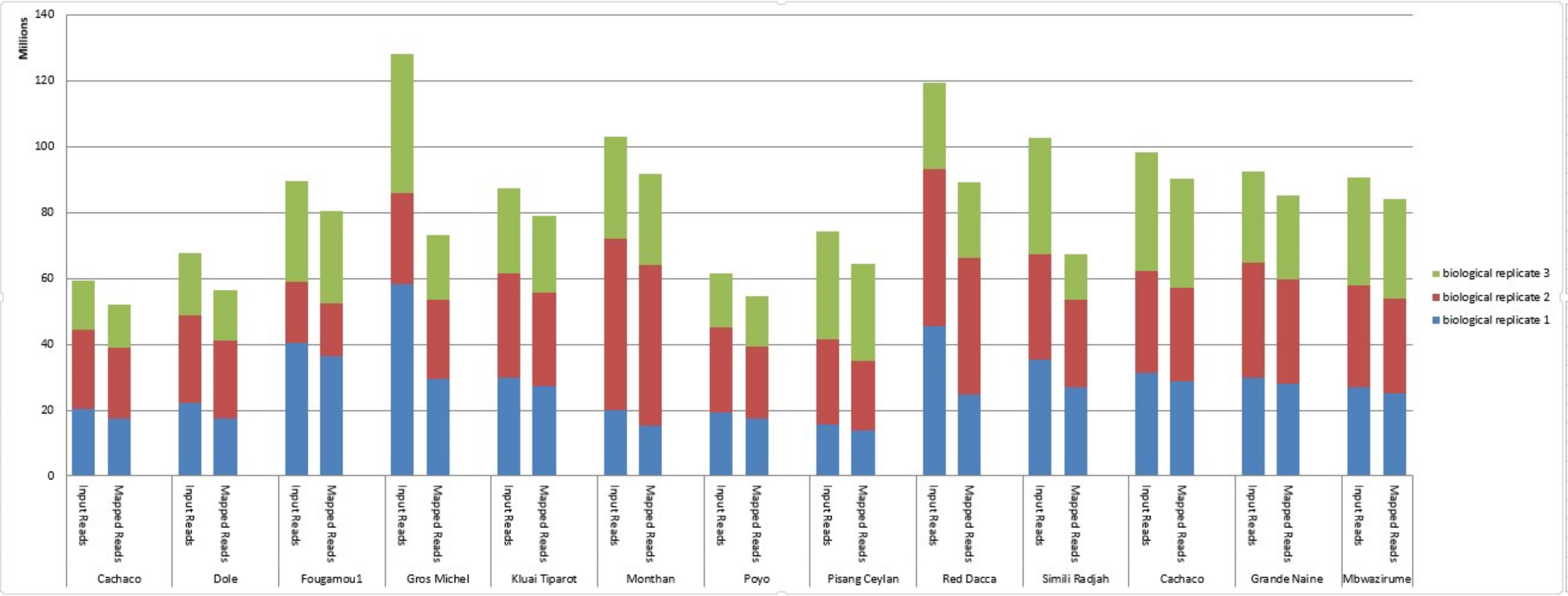

Supplement: Supplementary file 1 — Sequencing and genome mapping statistics for the 12 genotypes considered in the study. Since ‘Cachaco’ was present in both the experiments, it was represented twice. (PNG 1026 kb) [file 12864_2019_5618_MOESM1_ESM.png]

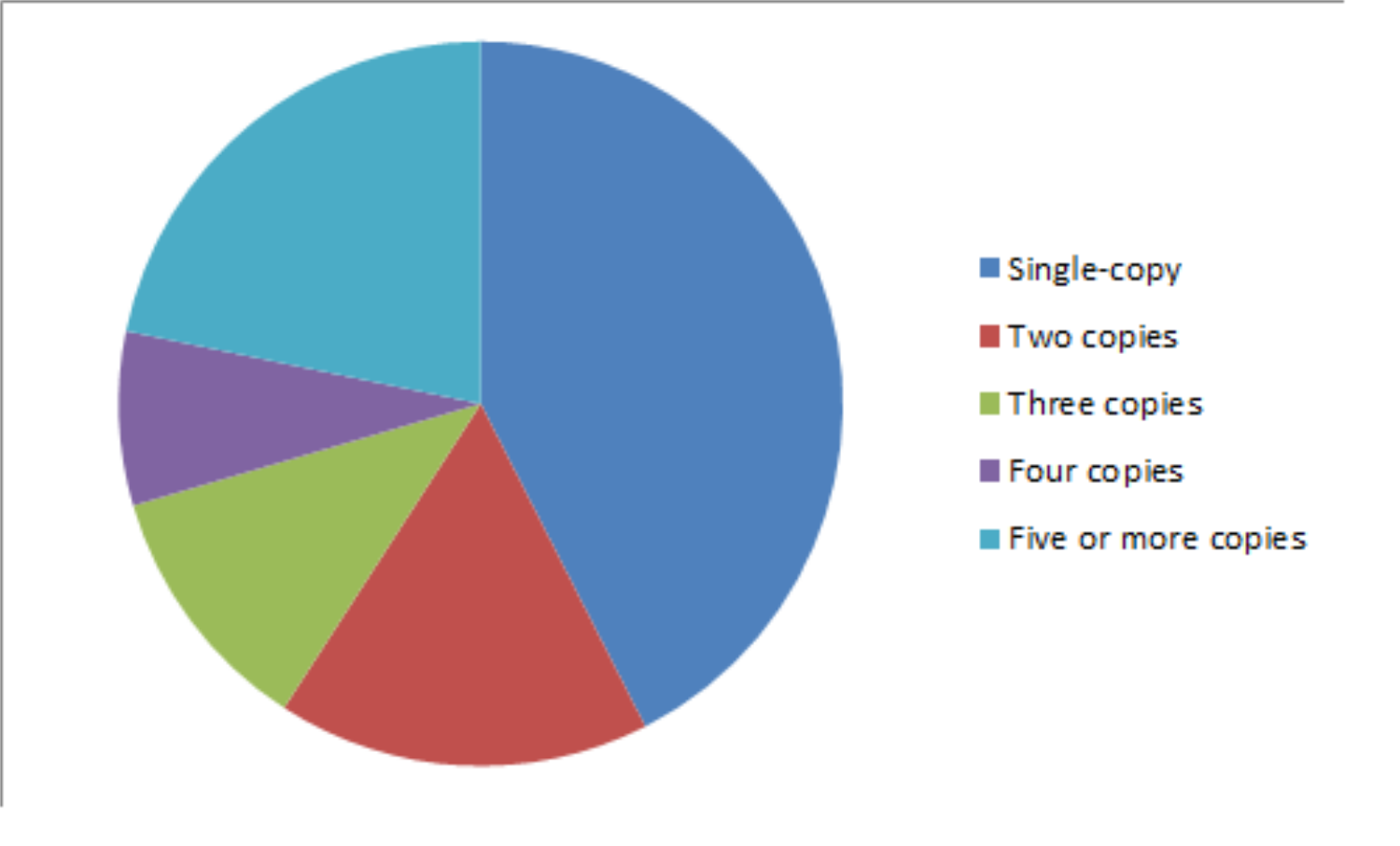

Supplement: Supplementary file 4 — Distribution of the 3674 DEGs by number of paralogs. (PNG 128 kb) [file 12864_2019_5618_MOESM4_ESM.png]

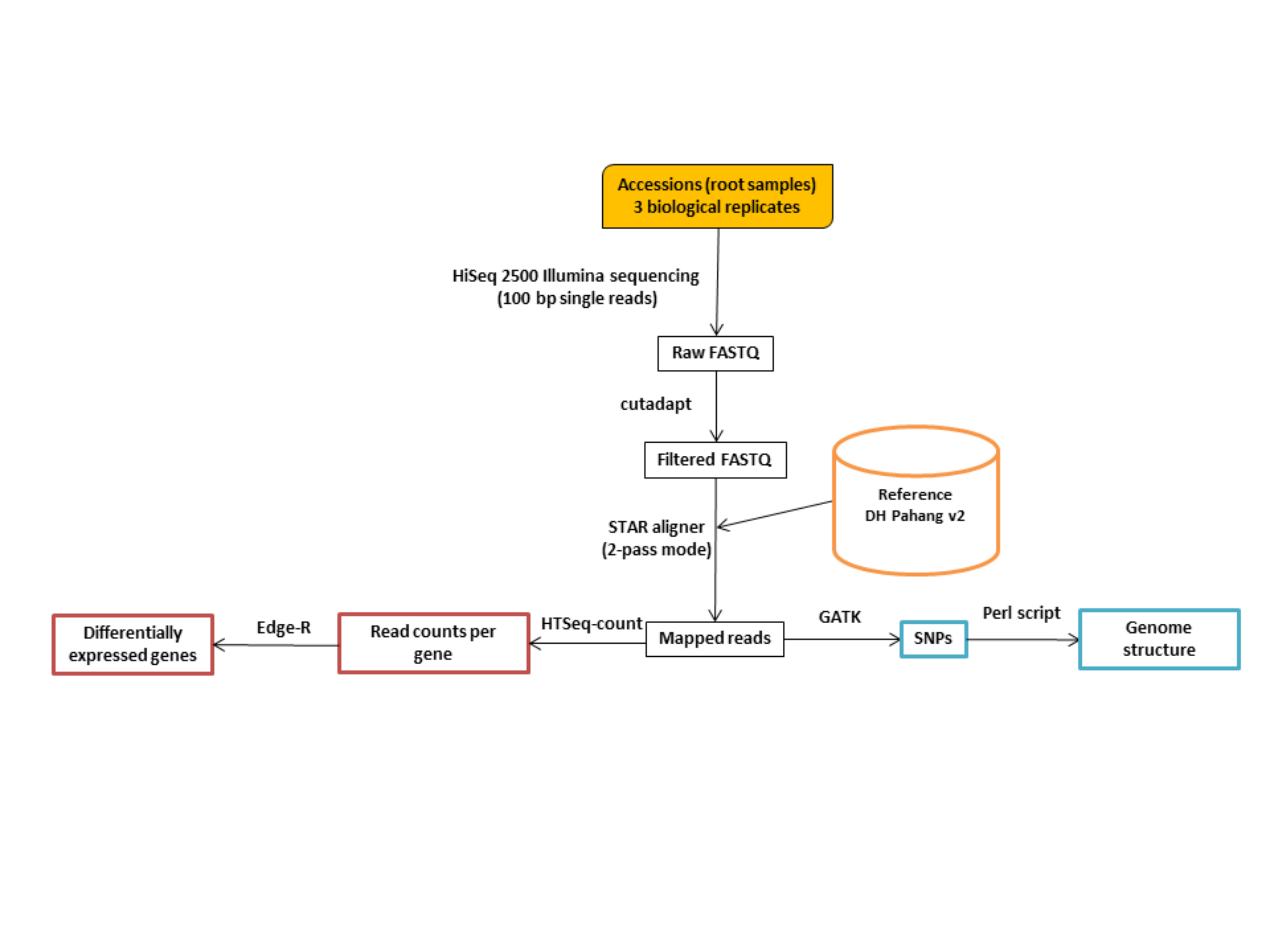

Supplement: Supplementary file 7 — Schematic view of the bioinformatics workflow for differential gene expression and genome structure identification. (PNG 310 kb) [file 12864_2019_5618_MOESM7_ESM.png]
