# Supplementary material for: Effect of paleopolyploidy and allopolyploidy on gene expression in banana
Source: BMC Genomics. 2019 Mar 27;20:244. doi: 10.1186/s12864-019-5618-0 (PMC6438041; doi:10.1186/s12864-019-5618-0)
Supplement: Supplementary file 2 — Chromosome intervals (defined on genes coordinates) showing B:A ratio deviating from the expected genomic constitution. CDM indicates the three cultivars sharing the same pattern of deviating regions (‘Cachaco’, ‘Dole’ and ‘Monthan’). (DOCX 16 kb) [file 12864_2019_5618_MOESM2_ESM.docx]

# **Additional file 2.**

| **Chromosome** | **Cultivars** | **Deviating ratio** | **Involved chromosome interval** |
| --- | --- | --- | --- |
| Chr04 | CDM | B2:A1=>B1:A2 | Ma04_g04070 - Ma04_g08760 |
| Chr04 | CDM+’Simili Radjah’ | B2:A1=>B3:A0 | Ma04_g27130 - Ma04_g40180 |
| Chr05 | ‘Simili Radjah’ | B2:A1=>B3:A0 | Ma05_g00010 - Ma05_g00250 |
| Chr05 | ‘Simili Radjah’ | B2:A1=>B3:A0 | Ma05_g31220 - Ma05_g32150 |
| Chr09 | CDM+’Simili Radjah’ | B2:A1=>B1:A2 | Ma09_g03240 - Ma09_g07160 |
| Chr11 | CDM | B2:A1=>B3:A0 | Ma11_g00010 - Ma11_g02580 |
| Chr11 | CDM+’Simili Radjah’ | B2:A1=>B3:A0 | Ma11_g09810 - Ma11_g25210 |
| Chr04 | ‘Fougamou1’ | B2:A1=>B3:A0 | Ma04_g00010 - Ma04_g08600 |
| Chr04 | ‘Fougamou1’ | B2:A1=>B3:A0 | Ma04_g32330 - Ma04_g36030 |
| Chr06 | ‘Fougamou1’ | B2:A1=>B1:A2 | Ma06_g33910 - Ma06_g38960 |
| Chr07 | ‘Fougamou1’ | B2:A1=>B3:A0 | Ma07_g26010 - Ma07_g29180 |
| Chr09 | ‘Fougamou1’ | B2:A1=>B1:A2 | Ma09_g28510 - Ma09_g31570 |
| Chr10 | ‘Fougamou1’ | B2:A1=>B3:A0 | Ma10_g12210 - Ma10_g31550 |
| Chr11 | ‘Fougamou1’ | B2:A1=>B1:A2 | Ma11_g00010 - Ma11_g04090 |
| Chr02 | ‘Kluai Tiparot’ | B2:A1=>B3:A0 | Ma02_g00010 - Ma02_g25170 |
| Chr03 | ‘Kluai Tiparot’ | B2:A1=>B3:A0 | Ma03_g00010 - Ma03_g10810 |
| Chr03 | ‘Kluai Tiparot’ | B2:A1=>B3:A0 | Ma03_g24810 - Ma03_g33710 |
| Chr04 | ‘Kluai Tiparot’ | B2:A1=>B3:A0 | Ma04_g00010 - Ma04_g35750 |
| Chr05 | ‘Kluai Tiparot’ | B2:A1=>B3:A0 | Ma05_g00010 - Ma05_g01260 |
| Chr05 | ‘Kluai Tiparot’ | B2:A1=>B3:A0 | Ma05_g30470 - Ma05_g32150 |
| Chr06 | ‘Kluai Tiparot’ | B2:A1=>B3:A0 | Ma06_g10310 - Ma06_g38960 |
| Chr07 | ‘Kluai Tiparot’ | B2:A1=>B3:A0 | Ma07_g00010 - Ma07_g19920 |
| Chr07 | ‘Kluai Tiparot’ | B2:A1=>B3:A0 | Ma07_g28740 - Ma07_g29180 |
| Chr08 | ‘Kluai Tiparot’ | B2:A1=>B3:A0 | Ma08_g00010 - Ma08_g34890 |
| Chr09 | ‘Kluai Tiparot’ | B2:A1=>B3:A0 | Ma09_g00010 - Ma09_g02610 |
| Chr09 | ‘Kluai Tiparot’ | B2:A1=>B3:A0 | Ma09_g07770 - Ma09_g13100 |
| Chr09 | ‘Kluai Tiparot’ | B2:A1=>B3:A0 | Ma09_g29230 - Ma09_g31570 |
| Chr10 | ‘Kluai Tiparot’ | B2:A1=>B3:A0 | Ma10_g13050 - Ma10_g31550 |
| Chr11 | ‘Kluai Tiparot’ | B2:A1=>B3:A0 | Ma11_g00010 - Ma11_g25210 |
| Chr03 | ‘Pisang Ceylan’ | B1:A2=>B2:A1 | Ma03_g30070 - Ma03_g33710 |
| Chr04 | ‘Pisang Ceylan’ | B1:A2=>B2:A1 | Ma04_g21550 - Ma04_g26820 |
| Chr04 | ‘Pisang Ceylan’ | B1:A2=>B0:A3 | Ma04_g33290 - Ma04_g40180 |
| Chr07 | ‘Pisang Ceylan’ | B1:A2=>B2:A1 | Ma07_g00010 - Ma09_g14670 |
| Chr08 | ‘Pisang Ceylan’ | B1:A2=>B2:A1 | Ma08_g34320 - Ma09_g34890 |
| Chr09 | ‘Pisang Ceylan’ | B1:A2=>B2:A1 | Ma09_g00010 - Ma09_g03230 |
| Chr09 | ‘Pisang Ceylan’ | B1:A2=>B2:A1 | Ma09_g07160 - Ma09_g11450 |
| Chr11 | ‘Pisang Ceylan’ | B1:A2=>B2:A1 | Ma11_g00010 - Ma11_g01170 |
| Chr11 | ‘Pisang Ceylan’ | B1:A2=>B0:A3 | Ma11_g01940 - Ma11_g02560 |

CDM = ‘Cachaco’, ‘Dole’ and ‘Monthan’ cultivars.
