# Supplementary material for: Effect of paleopolyploidy and allopolyploidy on gene expression in banana
Source: BMC Genomics. 2019 Mar 27;20:244. doi: 10.1186/s12864-019-5618-0 (PMC6438041; doi:10.1186/s12864-019-5618-0)
Supplement: Supplementary file 5 — Sample of genes differentially expressed in ‘Cachaco’ (ABB) compared to AAA cultivars (‘Mbwazirume’ and ‘Grande Naine’). (DOCX 18 kb) [file 12864_2019_5618_MOESM5_ESM.docx]

**Additional file 5**.

| **Gene name** | **Function** | **Genomic**  **ratio in Cachaco*** | **Paralogs** | **Validated** |
| --- | --- | --- | --- | --- |
| Ma00_g03260 | Heavy metal-associated isoprenylated plant protein 3 |  | 4 | no |
| Ma01_g06730 | Long chain acyl-CoA synthetase 9, chloroplastic |  | 3 | no |
| Ma01_g08040 | Photosynthetic NDH subunit of subcomplex B 3, chloroplastic |  | 0 | yes |
| Ma01_g08950 | Uncharacterized |  | 0 | yes |
| Ma01_g10510 | Non-specific lipid-transfer protein 1-like |  | 9 | no |
| Ma01_g14620 | cytochrome P450 71A1 |  | 1 | no |
| Ma01_g15740 | G-type lectin S-receptor-like serine/threonine-protein kinase At2g19130 |  | >10 | N/A |
| Ma02_g19970 | Uncharacterized |  | >10 | N/A |
| Ma03_g16090 | Homeobox-leucine zipper protein HOX19 |  | 3 | no |
| Ma03_g23410 | Putative disease resistance protein RGA1 |  | >10 | N/A |
| Ma04_g14520 | Uncharacterized |  | 0 | yes |
| Ma04_g15380 | Probable LRR receptor-like protein kinase At1g51890 |  | >10 | N/A |
| Ma04_g31060 | Dihydroflavonol-4-reductase-like | B3:A0 | 0 | yes |
| Ma04_g33910 | S-norcoclaurine synthase 2-like | B3:A0 | 0 | yes |
| Ma04_g34090 | 50S ribosomal protein L19-2, chloroplastic | B3:A0 | 1 | no |
| Ma04_g36430 | Cytosolic sulfotransferase 8-like | B3:A0 | 4 | yes |
| Ma04_g37240 | V-type proton ATPase subunit c''2 | B3:A0 | 2 | no |
| Ma04_g39940 | Autophagy-related protein 8C | B3:A0 | 6 | no |
| Ma04_g39940 | Autophagy-related protein 8C-like |  | 3 | no |
| Ma05_g02000 | NADH dehydrogenase [ubiquinone] iron-sulfur protein 8, mitochondrial~ TYKY |  | 1 | no |
| Ma05_g02010 | NADH-ubiquinone oxidoreductase  subunit 8 |  | 1 | no |
| Ma05_g09570 | Potassium channel AKT1 |  | 3 | no |
| Ma05_g15730 | protein PIN-LIKES 7-like |  | 3 | no |
| Ma05_g16660 | Synaptotagmin-2 |  | 1 | no |
| Ma05_g17890 | Cysteine synthase |  | 1 | no |
| Ma05_g18820 | Probable aldo-keto reductase 2 |  | 1 | no |
| Ma05_g27610 | 40S ribosomal protein S5 |  | 4 | no |
| Ma06_g01690 | Beta-hexosaminidase 3-like |  | 0 | yes |
| Ma06_g01690 | Oligopeptide transporter 5-like |  | >10 | N/A |
| Ma06_g05300 | Uncharacterized |  | 1 | no |
| Ma06_g07420 | Uncharacterized |  | 3 | no |
| Ma06_g21380 | Uncharacterized protein PHLOEM PROTEIN 2-LIKE A4 |  | 3 | no |
| Ma07_g08450 | Protein of unknown function (DUF2921) |  | 0 | yes |
| Ma07_g15390 | Elongation factor 1-alpha-like |  | >10 | N/A |
| Ma07_g19400 | Vacuolar amino acid transporter 1 |  | 0 | yes |
| Ma08_g09420 | Two-component response regulator ORR9 |  | 5 | no |
| Ma08_g14830 | Chorismate synthase 1, chloroplastic |  | 2 | no |
| Ma08_g31180 | Glutamate decarboxylase 4 |  | 1 | no |
| Ma09_g17380 | Cytochrome P450 71A9-like |  | 0 | yes |
| Ma09_g25370 | Beta-xylosidase/alpha-L-arabinofuranosidase 2-like |  | 0 | yes |
| Ma10_g08240 | Putative disease resistance protein RGA3 |  | >10 | N/A |
| Ma10_g19610 | Uncharacterized |  | 0 | yes |
| Ma10_g24290 | Transcription termination factor MTERF15, mitochondrial-like |  | >10 | N/A |
| Ma10_g28130 | Aminopeptidase M1 |  | 1 | no |
| Ma11_g00590 | MYB-like DNA-binding domain, SHAQKYF class | B3:A0 | 0 | yes |
| Ma11_g13680 | Calreticulin-3-like | B3:A0 | 1 | no |
| Ma11_g14110 | Ubiquitin thioesterase otubain-like | B3:A0 | 2 | no |
| Ma11_g14590 | Calmodulin-like | B3:A0 | 1 | yes |
| Ma11_g16110 | Vesicle-associated protein 1-2-like | B3:A0 | 3 | no |
| Ma11_g17510 | Basic leucine zipper and W2 domain-containing protein 2 | B3:A0 | 1 | no |
| Ma11_g19200 | Probable NADH dehydrogenase [ubiquinone] 1 alpha subcomplex subunit 5, mitochondrial~ At5g52840 | B3:A0 | 1 | no |
| Ma11_g19840 | MADS-box transcription factor 50-like | B3:A0 | 4 | no |
| Ma11_g20920 | Enolase | B3:A0 | 1 | no |
| Ma11_g21220 | Chorismate synthase, chloroplastic-like | B3:A0 | 2 | no |
| Ma11_g22410 | Ribonuclease II, chloroplastic/mitochondrial | B3:A0 | 0 | yes |
| Ma11_g24100 | 40S ribosomal protein S12 | B3:A0 | 4 | no |

* Genomic ratio is indicated when different from the expected B2:A1.
