# Supplementary material for: Effect of paleopolyploidy and allopolyploidy on gene expression in banana
Source: BMC Genomics. 2019 Mar 27;20:244. doi: 10.1186/s12864-019-5618-0 (PMC6438041; doi:10.1186/s12864-019-5618-0)
Supplement: Supplementary file 6 — Histograms representing the paralog included expression (normalized read count, Y axis) for 58 genes having significant higher expression in ‘Cachaco’ (Ca) than in ‘Grande Naine’ (GN) and ‘Mbwazirume’ (Mb). Blue color represents expression level of DEG, other colors expression level of respective paralogs. (PPTX 146 kb) [file 12864_2019_5618_MOESM6_ESM.pptx]

## Slide 1
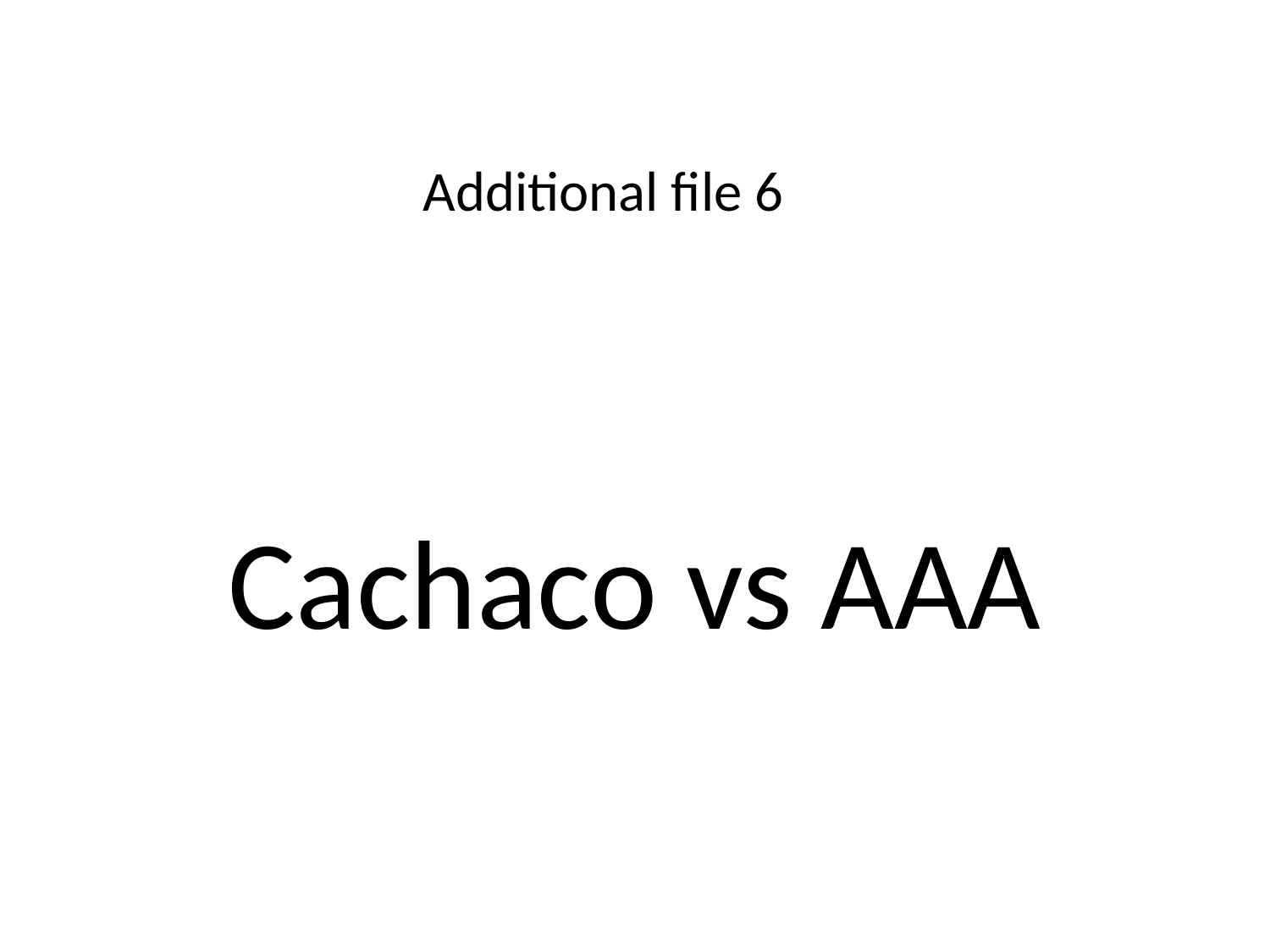

Additional file 6
# Cachaco vs AAA

## Slide 2
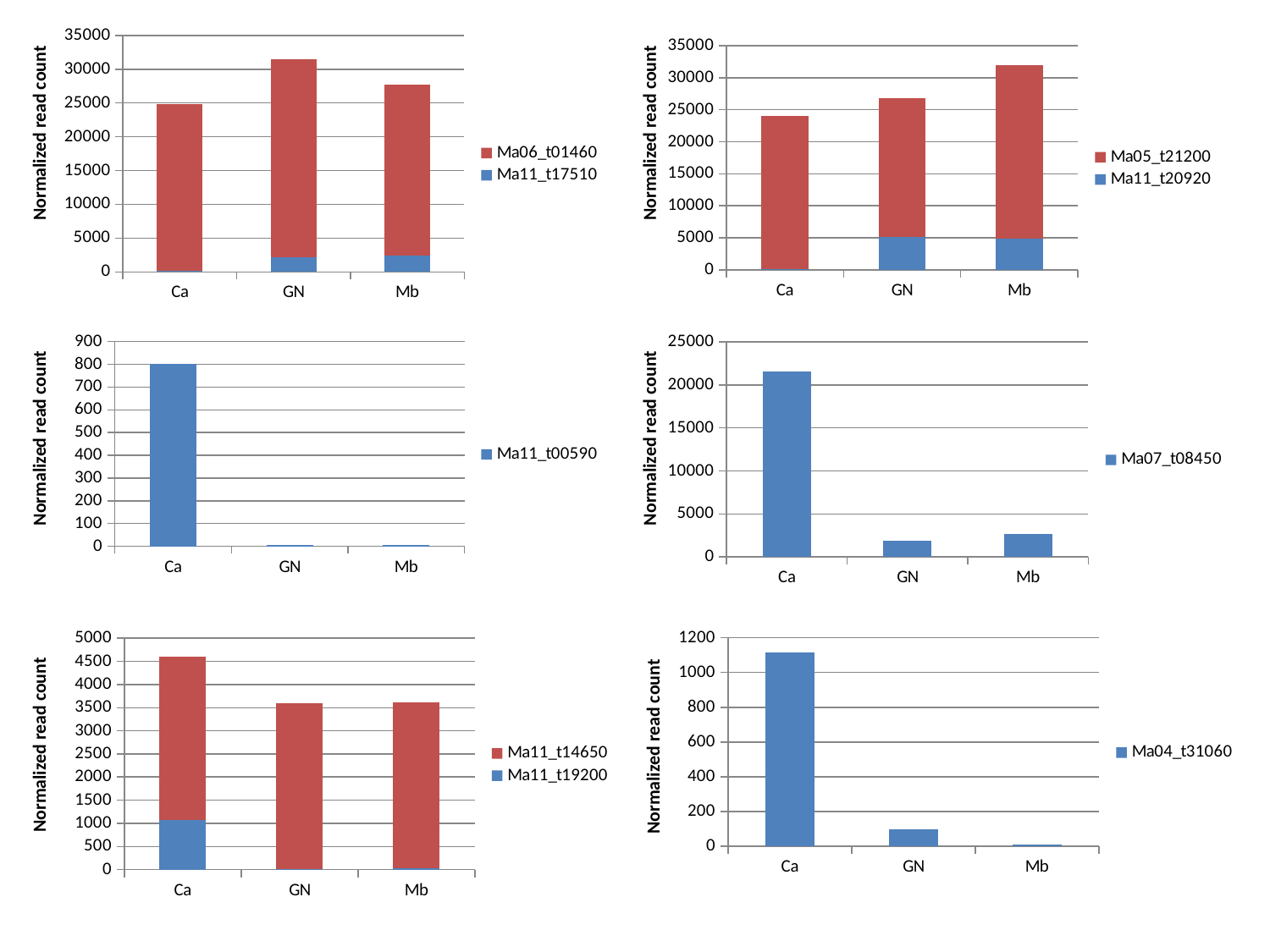

### Chart
| Category | Ma11_t17510 | Ma06_t01460 |
|---|---|---|
| Ca | 126.66431195971322 | 24743.38171917499 |
| GN | 2189.7801058680147 | 29296.51964159195 |
| Mb | 2387.951744354595 | 25344.195961292702 |
### Chart
| Category | Ma11_t20920 | Ma05_t21200 |
|---|---|---|
| Ca | 154.28702489575076 | 23922.62236252101 |
| GN | 5078.100168742745 | 21779.212898421814 |
| Mb | 4838.960806926208 | 27142.257290815585 |Normalized read count
Normalized read count
### Chart
| Category | Ma11_t00590 |
|---|---|
| Ca | 803.3394861393399 |
| GN | 6.214289750006903 |
| Mb | 5.798643610602542 |
### Chart
| Category | Ma07_t08450 |
|---|---|
| Ca | 21577.65535793195 |
| GN | 1879.6218492924943 |
| Mb | 2666.4934099816514 |Normalized read count
Normalized read count
### Chart
| Category | Ma11_t19200 | Ma11_t14650 |
|---|---|---|
| Ca | 1076.2970197272018 | 3523.4881066188764 |
| GN | 14.757444903047087 | 3576.9460105346243 |
| Mb | 33.140210666836936 | 3578.4729636675665 |
### Chart
| Category | Ma04_t31060 |
|---|---|
| Ca | 1113.3195076179895 |
| GN | 96.878563401644 |
| Mb | 8.92918646914828 |Normalized read count
Normalized read count

## Slide 3
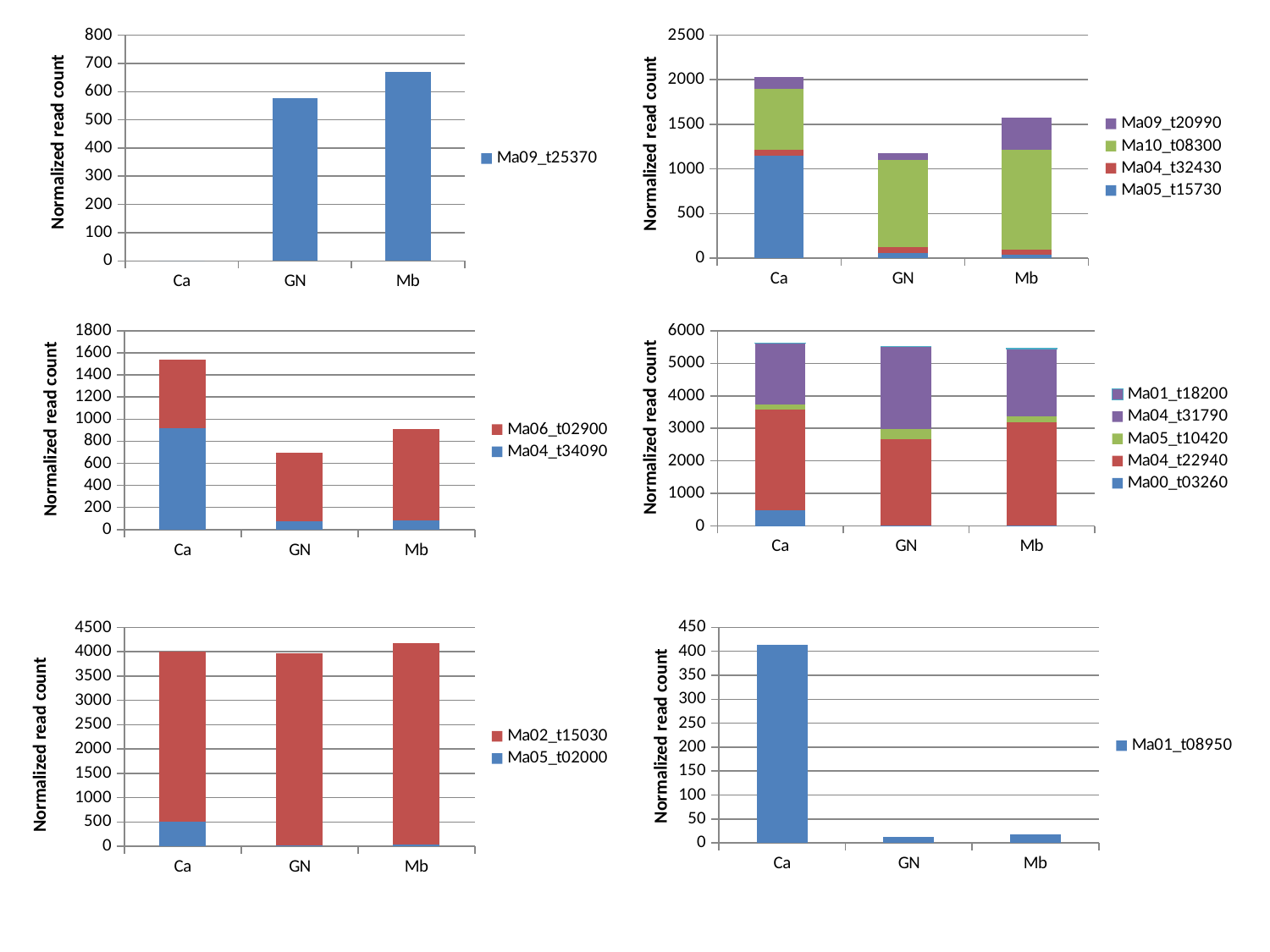

### Chart
| Category | Ma09_t25370 |
|---|---|
| Ca | 0.0 |
| GN | 577.5629324223206 |
| Mb | 668.4887701316654 |
### Chart
| Category | Ma05_t15730 | Ma04_t32430 | Ma10_t08300 | Ma09_t20990 |
|---|---|---|---|---|
| Ca | 1145.0536856284555 | 68.87607350722342 | 684.5197300870659 | 133.006136141073 |
| GN | 56.59211375265498 | 67.62110908191414 | 972.6948348312158 | 80.88622746326836 |
| Mb | 43.26148480357686 | 56.612360513451236 | 1116.8255391001287 | 359.78716172058193 |Normalized read count
Normalized read count
### Chart
| Category | Ma04_t34090 | Ma06_t02900 |
|---|---|---|
| Ca | 919.936492222037 | 615.0482631680829 |
| GN | 75.8363704147781 | 620.4113131857946 |
| Mb | 85.25889857977333 | 829.0247150648537 |
### Chart
| Category | Ma00_t03260 | Ma04_t22940 | Ma05_t10420 | Ma04_t31790 | Ma01_t18200 |
|---|---|---|---|---|---|
| Ca | 485.90258784621767 | 3092.1493679176547 | 165.59561483799985 | 1864.46028787966 | 6.632931306619144 |
| GN | 0.9874563420864756 | 2673.2191001373953 | 316.5683144370958 | 2517.4427736688112 | 12.89510755693202 |
| Mb | 10.086150265687323 | 3171.43497207567 | 177.6047083750273 | 2085.143158930338 | 27.210263247895348 |Normalized read count
Normalized read count
### Chart
| Category | Ma05_t02000 | Ma02_t15030 |
|---|---|---|
| Ca | 501.57620992063823 | 3494.5100500484796 |
| GN | 28.455003389854486 | 3937.5429733614683 |
| Mb | 40.83295680736933 | 4141.467201574841 |
### Chart
| Category | Ma01_t08950 |
|---|---|
| Ca | 413.8520470432991 |
| GN | 12.173115753990793 |
| Mb | 18.18951123644645 |Normalized read count
Normalized read count

## Slide 4
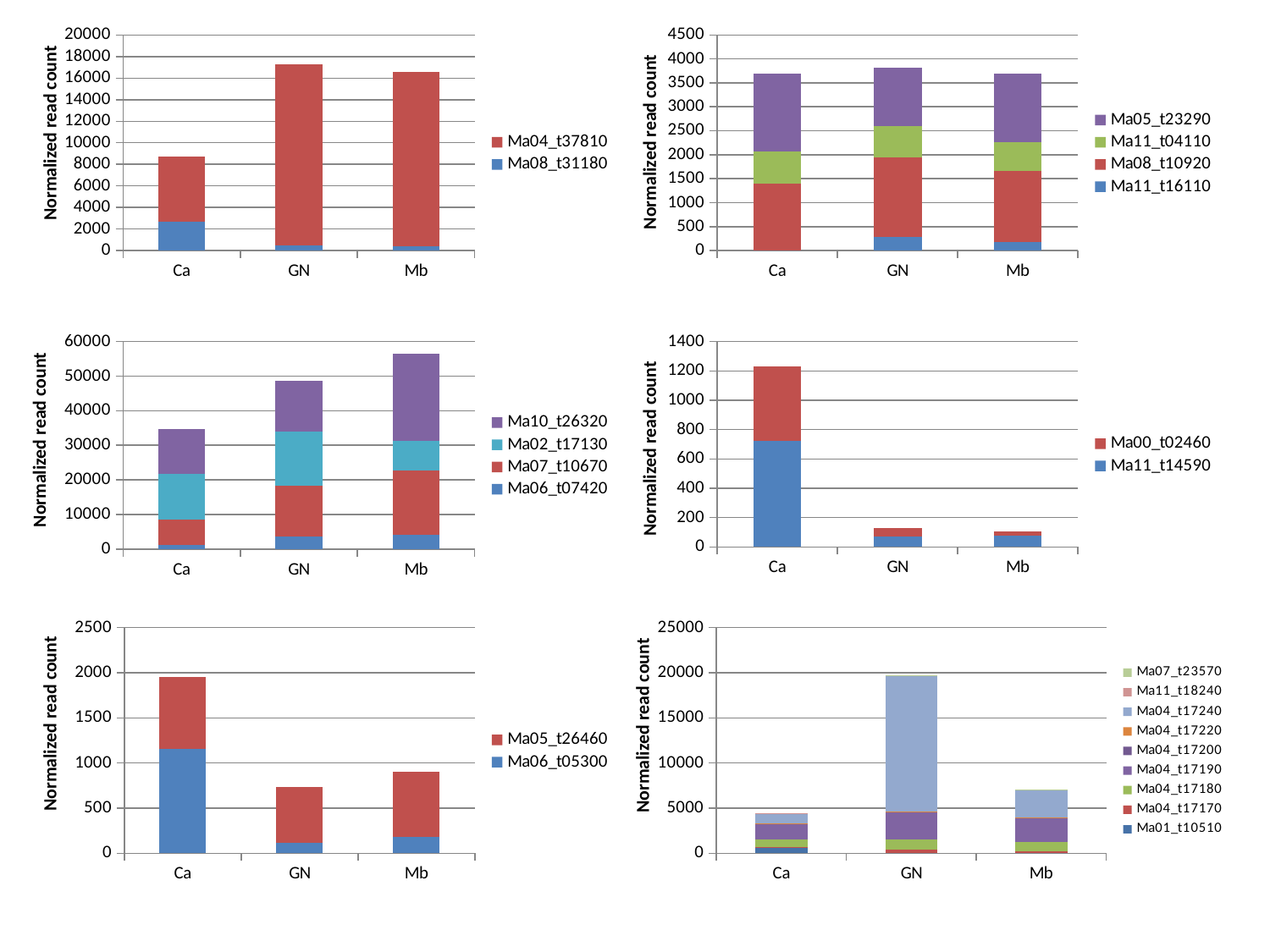

### Chart
| Category | Ma11_t16110 | Ma08_t10920 | Ma11_t04110 | Ma05_t23290 |
|---|---|---|---|---|
| Ca | 0.0 | 1397.282196831797 | 676.9451569437051 | 1627.499471262125 |
| GN | 280.3386874717363 | 1667.4135373449174 | 650.0795714826415 | 1212.3563248447706 |
| Mb | 186.24825236666862 | 1475.5461594068536 | 599.4159006229694 | 1423.5069619406336 |
### Chart
| Category | Ma08_t31180 | Ma04_t37810 |
|---|---|---|
| Ca | 2640.5968165136587 | 6040.029482668935 |
| GN | 506.2303029417752 | 16782.418033751 |
| Mb | 393.9438406997475 | 16163.665321622415 |Normalized read count
Normalized read count
### Chart
| Category | Ma06_t07420 | Ma07_t10670 | Ma02_t17130 | Ma10_t26320 |
|---|---|---|---|---|
| Ca | 1168.7672997720142 | 7250.554186956488 | 13202.963630332284 | 13136.960592028014 |
| GN | 3613.5578159647034 | 14764.416641395303 | 15715.994095714395 | 14544.656247608997 |
| Mb | 4197.064722693757 | 18407.502680368707 | 8773.082709244438 | 25026.409637556822 |
### Chart
| Category | Ma11_t14590 | Ma00_t02460 |
|---|---|---|
| Ca | 720.8934685391339 | 510.07361890021986 |
| GN | 73.01066388491935 | 56.214073875451845 |
| Mb | 78.44668399119456 | 25.804516462585532 |Normalized read count
Normalized read count
### Chart
| Category | Ma06_t05300 | Ma05_t26460 |
|---|---|---|
| Ca | 1154.9840546008206 | 796.4205508576872 |
| GN | 116.09628187541767 | 616.9273399116059 |
| Mb | 184.00459107274588 | 717.5748817697657 |
### Chart
| Category | Ma01_t10510 | Ma04_t17170 | Ma04_t17180 | Ma04_t17190 | Ma04_t17200 | Ma04_t17220 | Ma04_t17240 | Ma11_t18240 | Ma07_t23570 |
|---|---|---|---|---|---|---|---|---|---|
| Ca | 599.823031510238 | 102.3898127335725 | 837.061497289355 | 1721.1652067054406 | 24.15169196558336 | 48.172115563058185 | 1095.164578996811 | 21.286098199003007 | 0.0 |
| GN | 0.0 | 396.3610733300339 | 1105.7087293945783 | 3146.526354740931 | 3.2438916709665424 | 5.122287122643478 | 15002.312320418769 | 40.68629425365312 | 4.512870657760134 |
| Mb | 0.0 | 191.5522087770319 | 1042.2505447231524 | 2714.303194581289 | 2.2880139549469876 | 5.418556813492725 | 3123.8196295548837 | 11.574252360376253 | 4.998731750344484 |Normalized read count
Normalized read count

## Slide 5
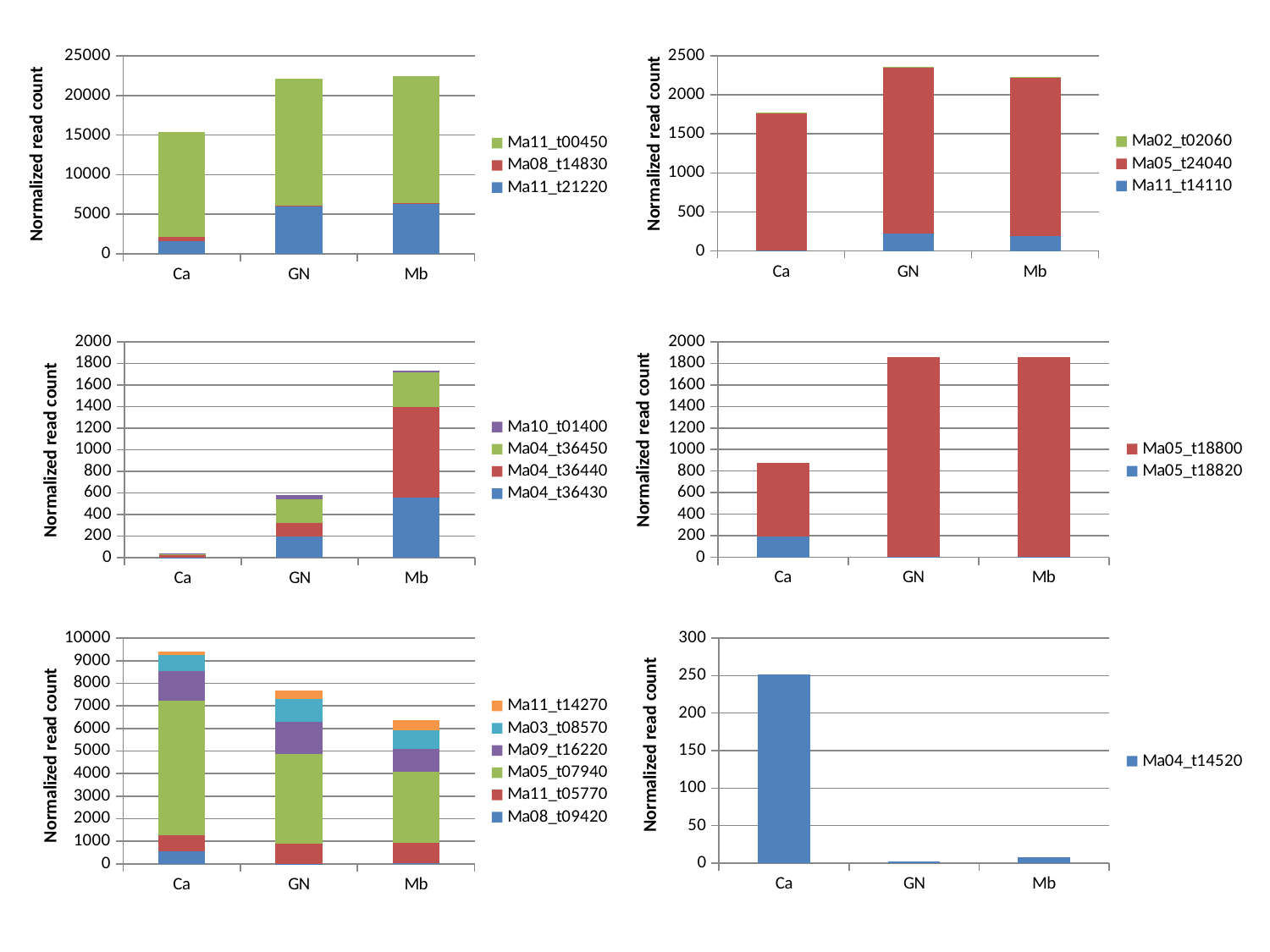

### Chart
| Category | Ma11_t21220 | Ma08_t14830 | Ma11_t00450 |
|---|---|---|---|
| Ca | 1632.9955371585024 | 561.6442651251086 | 13241.649330128032 |
| GN | 6024.622069858986 | 69.58799271330616 | 16051.528560198594 |
| Mb | 6396.082943323292 | 24.220234430610162 | 15962.561452964168 |
### Chart
| Category | Ma11_t14110 | Ma05_t24040 | Ma02_t02060 |
|---|---|---|---|
| Ca | 1.9658077742212723 | 1767.2220132934078 | 2.1063074429532955 |
| GN | 219.54080531504707 | 2134.2598494988015 | 2.0714299166689676 |
| Mb | 184.92486089073793 | 2034.8495199935849 | 7.9553537572104815 |Normalized read count
Normalized read count
### Chart
| Category | Ma04_t36430 | Ma04_t36440 | Ma04_t36450 | Ma10_t01400 |
|---|---|---|---|---|
| Ca | 1.1316559659164622 | 20.6455972796351 | 7.483587935071561 | 11.935346314059657 |
| GN | 192.3608382244258 | 131.69026565980516 | 213.72731874903494 | 39.061559406782706 |
| Mb | 553.2513938205725 | 845.1433566140779 | 314.76461381309787 | 22.01457578504229 |
### Chart
| Category | Ma05_t18820 | Ma05_t18800 |
|---|---|---|
| Ca | 194.4630683092521 | 682.9533219580317 |
| GN | 2.167947149164984 | 1856.522339261889 |
| Mb | 2.2223620507774635 | 1852.8708906030724 |Normalized read count
Normalized read count
### Chart
| Category | Ma04_t14520 |
|---|---|
| Ca | 251.6852862305156 |
| GN | 2.256435328880067 |
| Mb | 7.798136310740336 |
### Chart
| Category | Ma08_t09420 | Ma11_t05770 | Ma05_t07940 | Ma09_t16220 | Ma03_t08570 | Ma11_t14270 |
|---|---|---|---|---|---|---|
| Ca | 535.8591629686293 | 732.944659695082 | 5943.635669695492 | 1324.131363023013 | 717.0357111771224 | 166.9099374178861 |
| GN | 3.340408903462559 | 876.2650193291006 | 3987.1940329071836 | 1436.7761741921536 | 1005.9141135604643 | 381.37160395051643 |
| Mb | 25.839640333638144 | 898.0694124454601 | 3156.344429920103 | 1010.9802956798978 | 813.1393519299598 | 480.39024502381625 |Normalized read count
Normalized read count

## Slide 6
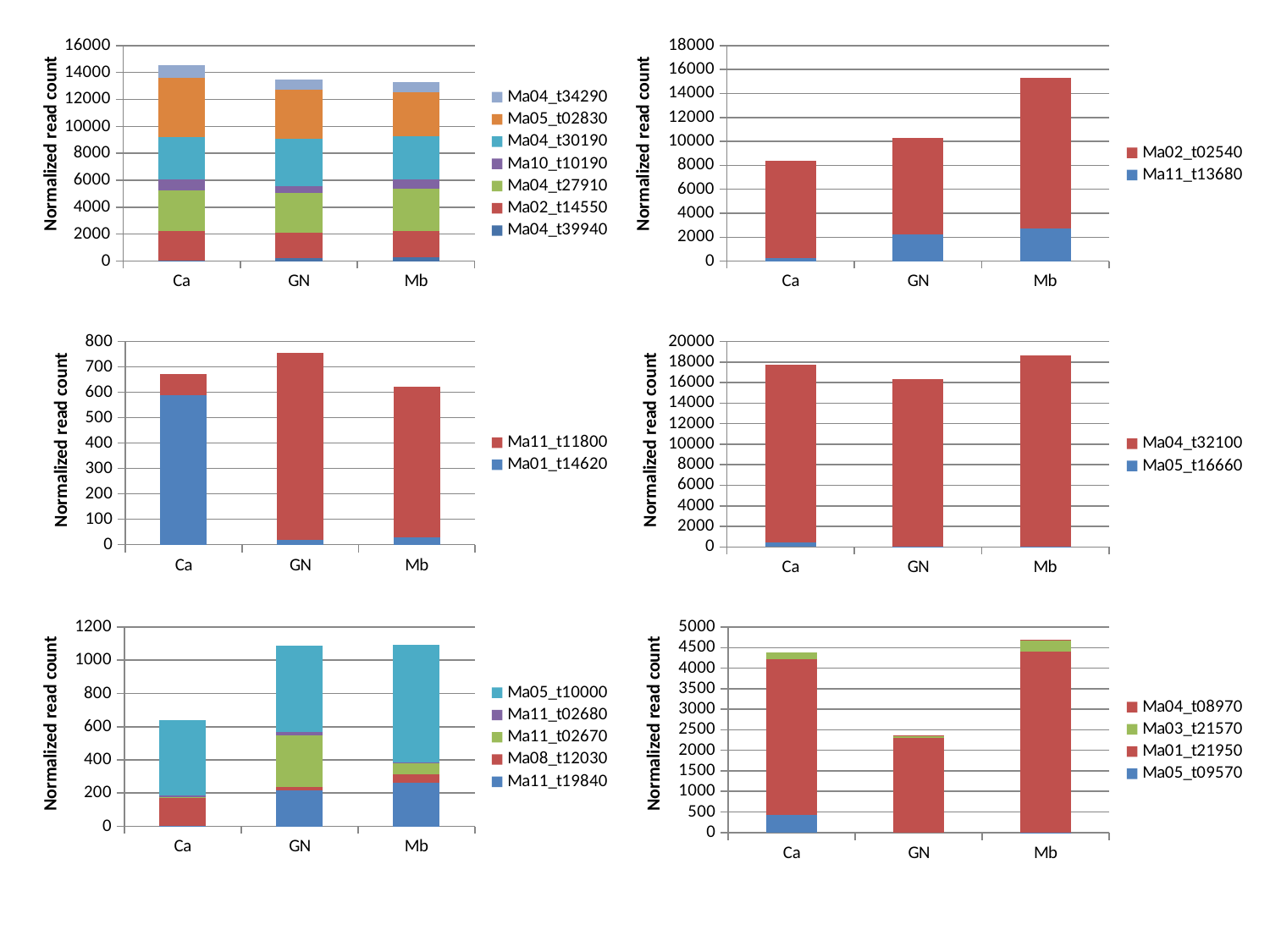

### Chart
| Category | Ma04_t39940 | Ma02_t14550 | Ma04_t27910 | Ma10_t10190 | Ma04_t30190 | Ma05_t02830 | Ma04_t34290 |
|---|---|---|---|---|---|---|---|
| Ca | 18.146778614482734 | 2202.0498603133938 | 3002.277113686505 | 864.8828902688743 | 3136.071123720534 | 4401.000889008842 | 910.691862487723 |
| GN | 236.8003170989998 | 1898.6076838149515 | 2909.765703616171 | 509.46354593082935 | 3512.2732965649325 | 3653.028010935166 | 785.2010431642152 |
| Mb | 298.8412248303883 | 1947.1942689255643 | 3099.286219363071 | 696.0710716604179 | 3235.196951975497 | 3233.3000356116445 | 791.1512636840633 |
### Chart
| Category | Ma11_t13680 | Ma02_t02540 |
|---|---|---|
| Ca | 240.54349746408616 | 8165.4367493832 |
| GN | 2263.3899871853378 | 8030.261978463556 |
| Mb | 2721.3919573865724 | 12579.214258584787 |Normalized read count
Normalized read count
### Chart
| Category | Ma01_t14620 | Ma11_t11800 |
|---|---|---|
| Ca | 588.7819829417756 | 82.55822568233252 |
| GN | 20.169283723178616 | 736.6501712123752 |
| Mb | 28.284871735055276 | 594.7143635734412 |
### Chart
| Category | Ma05_t16660 | Ma04_t32100 |
|---|---|---|
| Ca | 424.55115562695744 | 17282.98662236485 |
| GN | 2.256435328880067 | 16334.355121415238 |
| Mb | 3.4449777514860305 | 18606.12766228412 |Normalized read count
Normalized read count
### Chart
| Category | Ma11_t19840 | Ma08_t12030 | Ma11_t02670 | Ma11_t02680 | Ma05_t10000 |
|---|---|---|---|---|---|
| Ca | 1.1316559659164622 | 167.90962881830265 | 5.897423322663817 | 9.21388897597339 | 456.3723538048081 |
| GN | 215.89084312785198 | 20.963705600273055 | 310.55297437310486 | 18.64484099036201 | 522.7873187116568 |
| Mb | 263.045576322733 | 52.073192092293425 | 67.05498881440485 | 2.2223620507774635 | 709.9275989781868 |
### Chart
| Category | Ma05_t09570 | Ma01_t21950 | Ma03_t21570 | Ma04_t08970 |
|---|---|---|---|---|
| Ca | 431.96620111402274 | 3790.742359608402 | 156.4047017102073 | 0.0 |
| GN | 0.0 | 2300.7716575973736 | 54.52674114842564 | 5.500326999846609 |
| Mb | 1.0653982542384206 | 4398.745559608237 | 296.0763887970804 | 3.064890954376214 |Normalized read count
Normalized read count

## Slide 7
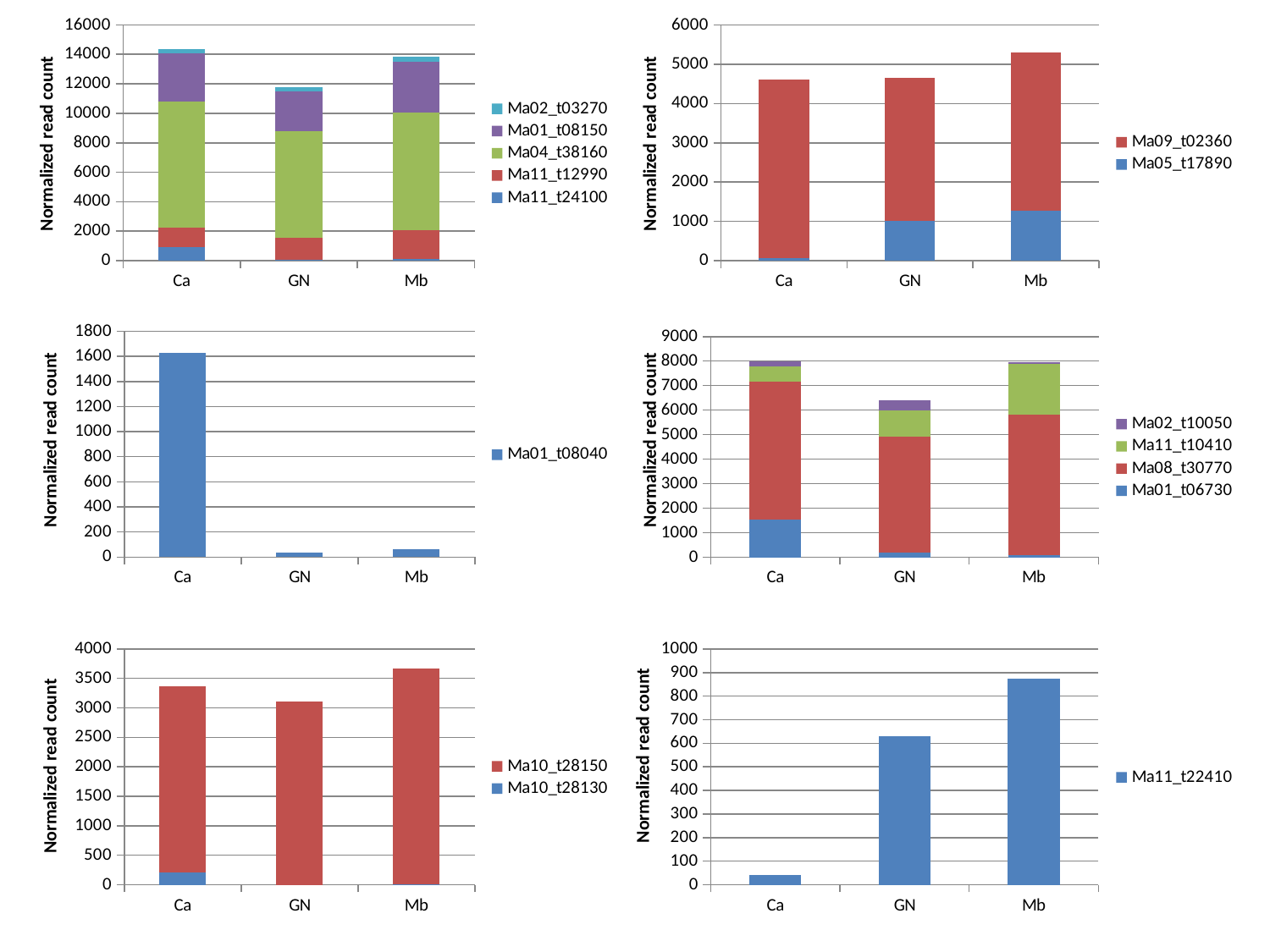

### Chart
| Category | Ma11_t24100 | Ma11_t12990 | Ma04_t38160 | Ma01_t08150 | Ma02_t03270 |
|---|---|---|---|---|---|
| Ca | 900.837857688918 | 1355.543717666854 | 8525.490050133034 | 3262.511797045755 | 325.4309803631611 |
| GN | 47.10787343299743 | 1506.0764809174768 | 7237.261212970743 | 2695.7604965788323 | 282.5625272998273 |
| Mb | 134.25165802914978 | 1960.5965418246765 | 7978.334995332147 | 3422.9254852350223 | 323.9402923093688 |
### Chart
| Category | Ma05_t17890 | Ma09_t02360 |
|---|---|---|
| Ca | 74.62778771682996 | 4526.040430112836 |
| GN | 1008.4881592998286 | 3652.6814409465583 |
| Mb | 1263.4346765017535 | 4032.162595788842 |Normalized read count
Normalized read count
### Chart
| Category | Ma01_t08040 |
|---|---|
| Ca | 1630.9758001895607 |
| GN | 35.60234925369782 |
| Mb | 61.0968228810446 |
### Chart
| Category | Ma01_t06730 | Ma08_t30770 | Ma11_t10410 | Ma02_t10050 |
|---|---|---|---|---|
| Ca | 1521.5494053241528 | 5627.590444171888 | 625.1692116559395 | 213.59943053635607 |
| GN | 182.84465029276936 | 4749.749929589938 | 1061.050299247744 | 422.88705592151916 |
| Mb | 97.80698365208737 | 5707.260077932897 | 2084.6906726010548 | 60.89986716853602 |Normalized read count
Normalized read count
### Chart
| Category | Ma10_t28130 | Ma10_t28150 |
|---|---|---|
| Ca | 215.19444135285113 | 3151.6698308315717 |
| GN | 0.0 | 3107.183218107761 |
| Mb | 3.064890954376214 | 3662.6830606984204 |
### Chart
| Category | Ma11_t22410 |
|---|---|
| Ca | 41.59233547664392 |
| GN | 628.551665340528 |
| Mb | 873.8779379585937 |Normalized read count
Normalized read count

## Slide 8
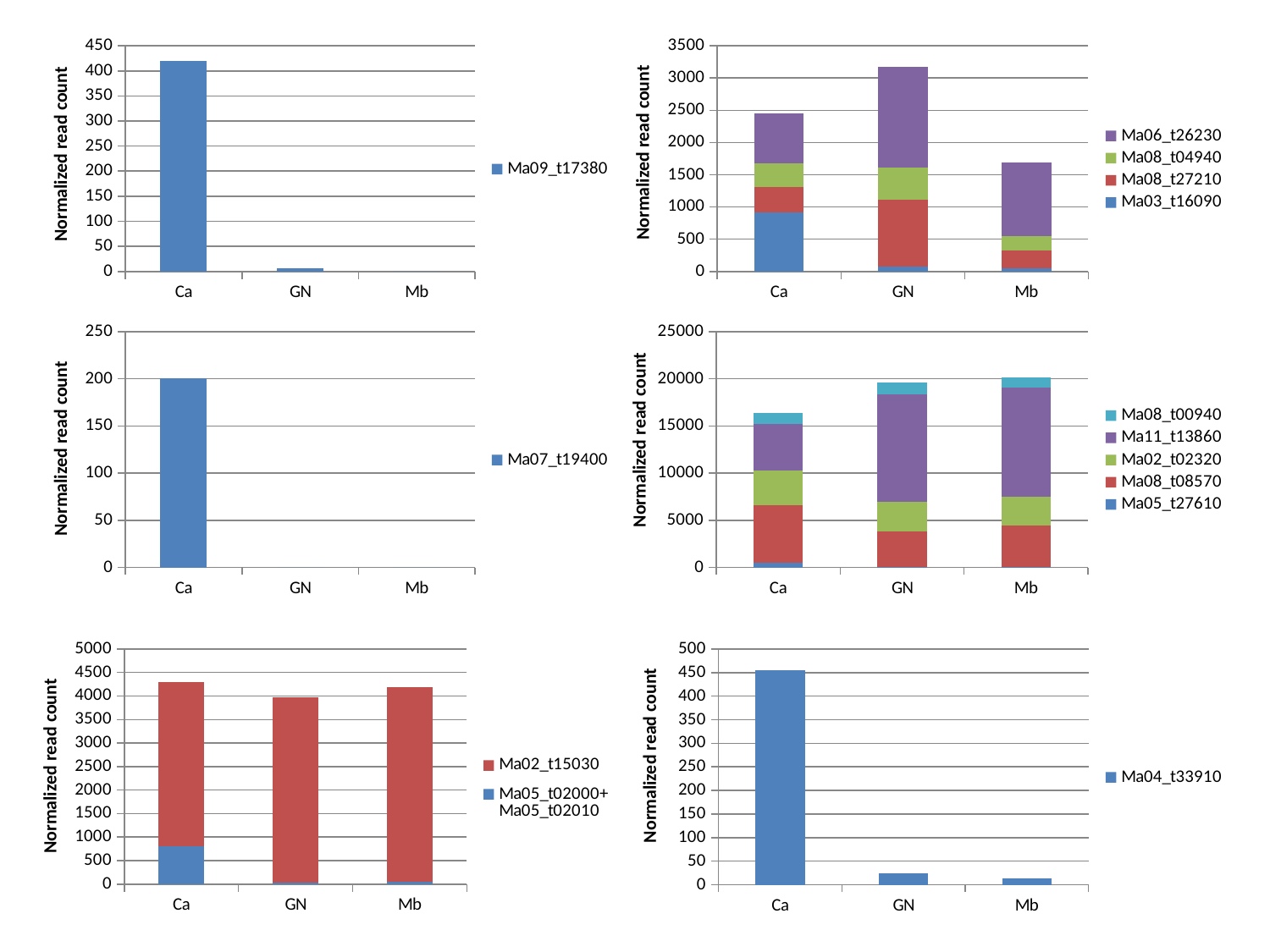

### Chart
| Category | Ma09_t17380 |
|---|---|
| Ca | 419.75839056573113 |
| GN | 6.479754289152151 |
| Mb | 0.0 |
### Chart
| Category | Ma03_t16090 | Ma08_t27210 | Ma08_t04940 | Ma06_t26230 |
|---|---|---|---|---|
| Ca | 914.5083616900871 | 392.1408870740188 | 365.04922724781295 | 778.1827302195213 |
| GN | 76.30289847169631 | 1032.1433583973046 | 497.1730933039214 | 1571.542119614802 |
| Mb | 55.16687546210301 | 271.81870706207997 | 224.3007989804998 | 1144.8010760954044 |Normalized read count
Normalized read count
### Chart
| Category | Ma07_t19400 |
|---|---|
| Ca | 200.14148971874408 |
| GN | 0.0 |
| Mb | 0.0 |
### Chart
| Category | Ma05_t27610 | Ma08_t08570 | Ma02_t02320 | Ma11_t13860 | Ma08_t00940 |
|---|---|---|---|---|---|
| Ca | 486.739811027968 | 6127.919659793489 | 3693.014673106346 | 4858.9371011961375 | 1190.1684726198334 |
| GN | 28.83304326705762 | 3791.884215816295 | 3133.8983743392073 | 11437.635666519107 | 1197.1720178590444 |
| Mb | 25.551119078828947 | 4398.178672360966 | 3111.2527867023605 | 11506.959238991752 | 1086.687464817851 |Normalized read count
Normalized read count
### Chart
| Category | Ma05_t02000+ Ma05_t02010 | Ma02_t15030 |
|---|---|---|
| Ca | 798.0539610485831 | 3494.5100500484796 |
| GN | 35.84175489415896 | 3937.5429733614683 |
| Mb | 51.07632451952679 | 4141.467201574841 |
### Chart
| Category | Ma04_t33910 |
|---|---|
| Ca | 454.6686653718492 |
| GN | 23.42120444692609 |
| Mb | 13.151041220063536 |Normalized read count
Normalized read count

## Slide 9
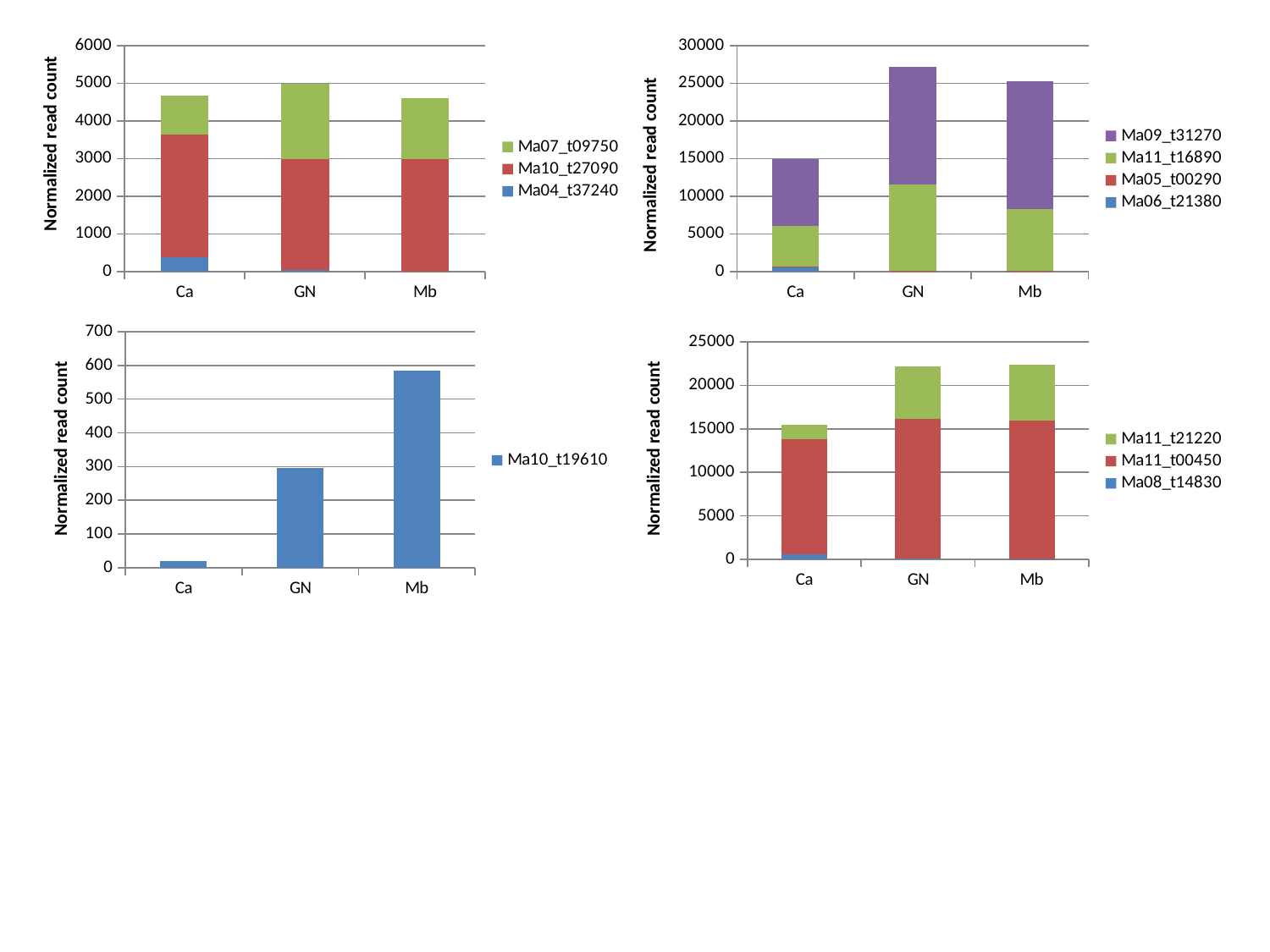

### Chart
| Category | Ma04_t37240 | Ma10_t27090 | Ma07_t09750 |
|---|---|---|---|
| Ca | 386.4434376769633 | 3247.6025967436062 | 1036.4761449384507 |
| GN | 40.107190858677015 | 2958.006090212657 | 1982.480200429959 |
| Mb | 0.0 | 2988.0501658002313 | 1628.4883797999978 |
### Chart
| Category | Ma06_t21380 | Ma05_t00290 | Ma11_t16890 | Ma09_t31270 |
|---|---|---|---|---|
| Ca | 584.7048953854501 | 9.135386731533575 | 5402.314868999939 | 8937.420071618686 |
| GN | 68.5862814068743 | 36.13130659164701 | 11499.115304572573 | 15605.088620483148 |
| Mb | 27.39339433249659 | 15.727576429819718 | 8244.845885075629 | 16949.57616013955 |Normalized read count
Normalized read count
### Chart
| Category | Ma10_t19610 |
|---|---|
| Ca | 19.43543906927882 |
| GN | 295.30688599513803 |
| Mb | 583.3479756620783 |
### Chart
| Category | Ma08_t14830 | Ma11_t00450 | Ma11_t21220 |
|---|---|---|---|
| Ca | 561.6442651251086 | 13241.649330128032 | 1632.9955371585024 |
| GN | 69.58799271330616 | 16051.528560198594 | 6024.622069858986 |
| Mb | 24.220234430610162 | 15962.561452964168 | 6396.082943323292 |Normalized read count
Normalized read count
